# A quantitative image analysis pipeline for the characterization of filamentous fungal morphologies as a tool to uncover targets for morphology engineering: a case study using *aplD* in *Aspergillus niger*

**DOI:** 10.1186/s13068-019-1473-0

**Published:** 2019-06-15

**Authors:** Timothy C. Cairns, Claudia Feurstein, Xiaomei Zheng, Ping Zheng, Jibin Sun, Vera Meyer

**Affiliations:** 1 Tianjin Institute of Industrial Biotechnology, Chinese Academy of Sciences, Tianjin, 300308 People’s Republic of China; 2 Key Laboratory of Systems Microbial Biotechnology, Chinese Academy of Sciences, Tianjin, 300308 People’s Republic of China; 3 Department of Applied and Molecular Microbiology, Institute of Biotechnology, Technische Universität Berlin, 13355 Berlin, Germany; 4 University of Chinese Academy of Sciences, 100049 Beijing, China

**Keywords:** Morphology engineering, *Aspergillus niger*, Image analysis, Pellet, Dispersed mycelia, Polar growth, Endocytosis, Tet-on, CRISPR, Protein secretion

## Abstract

**Background:**

Fungal fermentation is used to produce a diverse repertoire of enzymes, chemicals, and drugs for various industries. During submerged cultivation, filamentous fungi form a range of macromorphologies, including dispersed mycelia, clumped aggregates, or pellets, which have critical implications for rheological aspects during fermentation, gas/nutrient transfer, and, thus, product titres. An important component of strain engineering efforts is the ability to quantitatively assess fungal growth phenotypes, which will drive novel leads for morphologically optimized production strains.

**Results:**

In this study, we developed an automated image analysis pipeline to quantify the morphology of pelleted and dispersed growth (MPD) which rapidly and reproducibly measures dispersed and pelleted macromorphologies from any submerged fungal culture. It (i) enables capture and analysis of several hundred images per user/day, (ii) is designed to quantitatively assess heterogeneous cultures consisting of dispersed and pelleted forms, (iii) gives a quantitative measurement of culture heterogeneity, (iv) automatically generates key Euclidian parameters for individual fungal structures including particle diameter, aspect ratio, area, and solidity, which are also assembled into a previously described dimensionless morphology number MN, (v) has an in-built quality control check which enables end-users to easily confirm the accuracy of the automated calls, and (vi) is easily adaptable to user-specified magnifications and macromorphological definitions. To concomitantly provide proof of principle for the utility of this image analysis pipeline, and provide new leads for morphologically optimized fungal strains, we generated a morphological mutant in the cell factory *Aspergillus niger* based on CRISPR-Cas technology. First, we interrogated a previously published co-expression networks for *A. niger* to identify a putative gamma-adaptin encoding gene (*aplD*) that was predicted to play a role in endosome cargo trafficking. Gene editing was used to generate a conditional *aplD* expression mutant under control of the titratable Tet-on system. Reduced *aplD* expression caused a hyperbranched growth phenotype and diverse defects in pellet formation with a putative increase in protein secretion. This possible protein hypersecretion phenotype could be correlated with increased dispersed mycelia, and both decreased pellet diameter and MN.

**Conclusion:**

The MPD image analysis pipeline is a simple, rapid, and flexible approach to quantify diverse fungal morphologies. As an exemplar, we have demonstrated that the putative endosomal transport gene *aplD* plays a crucial role in *A. niger* filamentous growth and pellet formation during submerged culture. This suggests that endocytic components are underexplored targets for engineering fungal cell factories.

**Electronic supplementary material:**

The online version of this article (10.1186/s13068-019-1473-0) contains supplementary material, which is available to authorized users.

## Background

Filamentous fungi are utilized as microbial cell factories for the production of proteins, acids, and secondary metabolites [1]. Many of these molecules constitute multi-billion-dollar industries, with their value expected to increase due to a shift from a petroleum to bio-based world-wide economy [2–4]. For example, fungal enzyme cocktails containing cellulases, hemicellulases, and ligninases are used to convert waste plant material to fermentable sugars, which are subsequently used as substrates in biofuel production with an estimated annual value of over $4.5 billion [1]. In addition, there is growing interest in the use of filamentous fungi for bioethanol fermentation due to their capability to grow on a wide range of sugars, and their high tolerance to many inhibitory molecules produced from hydrolysed plant biomass [5–7].

Filamentous fungi undergo complex morphological changes in submerged fermenters, and a growing body of evidence suggests that this has critical implications both for the titre of useful molecules and rheological aspects of fermentation media [8–10]. Consequently, strain engineering efforts which optimize morphological parameters for improved biotechnological applications are a major goal of applied fungal research [1, 2].

The fundamental cell of the filamentous fungal life cycle is thread like, multicellular hyphae, which branch to form a network termed a mycelium. In submerged culture, macromorphologies range from a dispersed mycelium [11, 12], to clumped aggregations of hyphae [13], to approximately spherical pellets of compact hyphae that are several millimetres in diameter [13, 14]. The predominant macromorphology produced in submerged culture is dependent on abiotic conditions and the specific fungal strain or species. Importantly, both positive and negative attributes from a process engineering perspective are apparent for each growth morphology, and consequently, labour and reagent intensive efforts are required to determine optimal morphologies for each desired molecule or production host.

For example, dispersed hyphae enhance production of certain acids (fumaric acid), proteins (amylases, neo-fructosyltransferase, and phytases) and secondary metabolites (penicillin) [15, 16]. However, the rheological consequences of dispersed growth elevate medium viscosity, which, in turn, can cause extensive temperature and nutrient concentration gradients within bioreactors as a result of heat and mass transfer limitations [17, 18]. Alternatively, clumps or pellets may be advantageous as the gas/liquid mass transfer of oxygen is improved, and the downstream separation of fungal biomass from fermentation media is straightforward when compared to dispersed mycelia. Pelleted macromorphologies favour production of certain molecules including citric acid [17, 19], glucoamylase [20], or polygalacturonidase [21]. A significant disadvantage of pelleted fungal macromorphologies is that internal areas may become hypoxic [22].

Given the complex and non-intuitive relationship between morphology and product titres, numerous efforts have recently been invested to control filamentous macromorphologies using both abiotic and molecular approaches. Abiotic approaches include modification of spore inoculum density [11], stir speed [23], oxygen enrichment [24], media pH [9], surfactants [25], carbon source [12], manganese availability [26, 27], addition of insoluble particles [28], osmolarity [29], in addition to others [14]. Alternatively, molecular strategies include UV or chemical mutagenesis approaches, or targeted disruption/deletion/knock down/conditional expression of genes required for polar growth, including cell wall biosynthesis, transcription factors, or GTPases [30–33].

A critical technical component of such studies is quantitative and high-throughput readouts of fungal macromorphology between control and optimized experimental cohorts. Due to existing challenges in establishing automated image analysis, many studies often describe morphological changes and optimisation in qualitative or semi-quantitative terms, whereby a single measurement of fungal macromorphology is determined (e.g., pellet diameter [30, 32, 33]). A second limitation is that image analysis is sufficiently labour intensive that only a small number of pellets are analysed. Consequently, such approaches may be vulnerable to inter-replicate variation and may not be sufficiently robust to detect subtle but significant modifications to fungal macromorphology that are, nevertheless, important from a process engineering perspective.

The previous work by Wucherpfennig and colleagues has demonstrated that multiple components of fungal pellets can be quantified and processed to generate a single dimensionless morphology number (MN) for individual fungal pellets [28, 29]. This quantitative approach measures pellet area, maximum diameter (Feret’s diameter), circularity (aspect ratio, i.e., the ratio of maximum diameter and minimum diameter), and solidity (a measurement of particle surface integrity), to generate an MN value between 0 (a theoretical one-dimensional line) and 1 (a perfect round sphere). The authors modified media osmolarity or added insoluble microparticles in *A. niger* submerged cultivations to control pellet morphologies [28, 29]. Using this approach, they were able to establish a clear inverse correlation between glucoamylase/β-fructofuranosidase production and pellet MN, indicating that smaller pellets/dispersed mycelia were optimal for protein secretion [28, 29]. Such quantitative descriptions of fungal macromorphology may enable improved understanding of the connection between filamentous growth and production.

Despite the clear utilities of such methods, several technical challenges are encountered when establishing such quantitative analysis pipelines for the end user: (i) lack of a flexible and simple image capture protocol; (ii) lack of automated image processing and analysis of pellet area, diameter, circularity, solidity, and MN; (iii) the challenge of working with heterogeneous cultures that contain both pelleted and dispersed morphologies, and (iv) lack of an intuitive analysis pipeline that enables end-users to view original images, processed images, and output data at an individual pellet level to aid quality control and interpretation of data.

To address these limitations, we developed a simple image capture protocol and automated analysis pipeline for quantification of fungal macromorphologies during submerged culture. Image analysis was designed to be compatible with diverse and simple image capture protocols (e.g., different cameras, microscopes, or magnifications). All parameters of individual pellet MNs are automatically calculated and detailed in an output file. Processed files and output data have individual fungal structures indexed, so that all data can be visually inspected by the end user. In addition, the percentage of pelleted morphologies and dispersed mycelia is calculated, enabling a simple quantitative measurement of heterogeneous cultures. The image analysis pipeline is available as a Java-based plugin for the ImageJ2/Fiji workstation. This protocol will enable flexible and high-throughput analysis of fungal morphology during submerged culture which will aid process engineering and strain development projects.

To test the quantitative image analysis pipeline, we reasoned that it was necessary to generate an *A. niger* mutant with defective filamentous growth and pellet morphology during submerged culture. Our rationale for this approach was that it would mimic comparable strain engineering efforts commonly used by fungal biotechnologists. A key process that underpins filamentous growth is endocytosis at the hyphal apex [34], yet the potential biotechnological applications of endocytic mutants have not been interrogated. To provide new potential leads for strain engineering efforts, we therefore generated conditional expression mutants in the acid, protein, and secondary metabolite production host *A. niger*. CRISPR-Cas9-mediated gene editing was used to place a titratable Tet-on cassette immediately upstream of a gene predicted to encode an gamma-adaptin (named AplD) that is associated with controlling filamentous morphology via endosomal vesicle trafficking [35]. Titration of gene expression using the tetracycline derivative doxycycline led to multiple intermediate phenotypes with hyperbranching, resistance to oxidative stress, elevated dispersed morphologies, and significant changes to pellet parameters and MN. These data suggest that endocytosis may be a promising strategy in future strain engineering efforts.

## Materials and methods

### Microbial strains

Fungal strains used in this study are given in Table 1. As progenitor isolate, we utilized strain MA70.15, which is deficient in the non-homologous end joining pathway to improve targeting of exogenous cassettes with the recipient genome, and also to reduce the occurrence of ectopic integration events [36]. All bacterial plasmids were propagated in *Escherichia coli* DH5α using 100 µg/mL ampicillin as selection.

**Table 1:** Fungal strains used in this study

Name	Genotype	Reference
MA70.15	*kusA::amdS, pyrG* ^−^	[36]
TC18.1	*kusA::amdS, pyrG*^−^*, PaplD::Tet*-*on*, *HygR*	This study
TC18.3	*kusA::amdS, pyrG*^−^*, PaplD::Tet*-*on*, *HygR*	This study

### Media

Strains of *A. niger* were grown at 30 °C in minimal medium (MM) [30] or complete medium (CM), consisting of MM supplemented with 1% yeast extract and 0.5% casamino acids [30]. In addition, Cit media used to model growth during citric acid production consisted of 3 g/L (NH_4_)_2_SO_4_, 3 g/L NaNO_3_, 0.5 g/L yeast extract, and 100 g/L sucrose, with the pH adjusted to 2.5 using HCl. All transformants were routinely grown in the presence of 100 μg/mL of hygromycin. All agar plates and liquid cultures were supplemented with 4 mM uridine.

### Co-expression analysis

The *A. niger aplD* co-expression network was retrieved from FungiDB [37]. From over 300 microarray experiments, only genes co-expressed above a Spearman correlation co-efficient of 0.7 were retrieved, giving a total of 109 genes with highly robust co-expression correlations [38]. The *aplD* network was interrogated for GO-enriched biological processes relative to the *A. niger* genome using default parameters in FungiDB, and those with Benjamini–Hochberg FDR corrected *p* values above 0.05 were reported [37, 38].

### Molecular techniques

All molecular techniques were performed according to the standard procedures described previously [30]. Plasmids were constructed using Gibson assembly [39] unless otherwise stated, and the transformation and genomic DNA extraction were performed as described elsewhere [40], with 5–10 µg/mL doxycycline (Dox) added to primary transformation plates and subculture media. Primers used in this study are given in Additional file 1.

### Genome editing

Annotated nucleic acid sequences detailing homologous loci of the single guide (sg) RNA, donor DNA, and verification primers at the *aplD* locus are given in Additional files 2 and 3. All plasmid sequences will be made available on reasonable request.

To design sgRNA with minimal chances of off-target cleavage, the 5′ UTR region of the AplD encoding gene (An01g02600) was screened using the sgRNAcas9 Software against *A. niger* genome (Ensemble) to generate a 20 bp targeting locus [41, 42]. sgRNA oligos 18An01g02600S3F and 18An01g02600S3R are homologous to this target site (Additional file 2), and were cloned into plasmid psgRNA6.0 [43] using BbsI to generate the derivative plasmid psg6.18. Generation of linear sgRNA constructs for *A. niger* transformation were generated by amplification using sequence verified sg6.18 plasmid as template and primers M13F and M13R as previously described [43]. This approach uses the *A. niger* 5S rRNA gene as promoter for sgRNA transcription [43].

For donor DNA fragments necessary to insert the Tet-on cassette at the *aplD* promoter, the Tet-on system from plasmid pFW22.1 [44] was PCR amplified and fused at the 3′ region of a hygromycin resistance cassette contained in plasmid pSilent-1 [45]. The resulting plasmid was sequence verified and termed pTC1.13. Generation of linear donor DNA constructs for homologous recombination of the Hyg-Tet-on fusion at the *aplD* promoter was generated by PCR using pTC1.13 as template and primers MH_An01g02600S3_ptrpc_F and MH_An01g02600S3_pmin_R. These primers amplify the Hyg-Tet-on cassette and contain 40 bp flanking regions to target the cassette to the *aplD* promoter locus (Additional files 1 and 2). A sequence of this donor construct is given in Additional file 3.

2 µg of purified Cas9 encoding plasmid pCas9-Hyg [Zheng et al., in preparation], was co-transformed with 2 µg purified sgRNA and donor constructs into *A. niger* MA70.15 protoplasts as previously described [43]. Primary transformants were selected on MM agar plates supplemented with 200 μg/mL hygromycin and 10 μg/mL Dox. Next, strains were purified twice on MM supplemented with 200 μg/mL hygromycin and 5–10 μg/mL Dox, after which genomic DNA was extracted from putative transformants. Insertion of the donor cassette at the *aplD* promoter was confirmed by diagnostic PCR using primers An01g02600-V-F and An01g02600-V-R (Additional file 2). PCR confirmed *A. niger* transformants were stored in 25% v/v glycerol at − 80 °C.

### Hyphal growth assays on solid medium

To generate a thin agar slice for light microscopic analysis, 8 mL MM agar was added to a 25 mL petri dish. Duplicate 10 µL volumes of 1 × 10^4^ spores/mL of mutant or control isolates were spotted onto the agar slice, air dried, and incubated at 30 °C for 18 h. Hyphae were imaged using a Zeiss Axio Cam Mrc5 light microscope. Hyphae were quantified for hyphal length and branch rate (length µm/number of branches) using ImageJ. Hyphal tip bursting in TC18.1 and TC18.3 mutants under 0 and 0.2 µg/mL Dox was recorded as a percentage of the total hyphae observed. Growth assays were repeated three times, with a minimum of 50 hyphae quantified per Dox concentration/strain.

### Phenotypic screens

*Aspergillus niger* conidia were harvested from 5-day cultivated CM agar plates. For conditional expression mutants, agar was supplemented with 100 μg/mL hygromycin and 20 μg/mL Dox. Spores were harvested in sterile water, filtered through Miracloth, and washed twice by centrifugation in 30 mL sterile water. Defined spore titres of *A. niger* isolates were spotted in 10 µL volumes of CM and MM agar plates, which were incubated for 7 days at 30 °C. Plates were inspected every 12 h and representative images were captured at indicated time points. Where specified, plates were supplemented with 1 or 10 mM H_2_O_2_. Phenotypic screens were conducted in technical triplicate.

### Culture conditions and image capture during submerged growth

For cultivation conditions approximating citric acid fermentation, 1 × 10^5^ spores/mL were inoculated into 20 mL Cit media in 100 mL Erlenmeyer flasks. Cultures were incubated at 34 °C, with 220 RPM, for 96 h, after which images were captured as described below.

Protein production in shake flasks was performed as previously described [30] with minor modifications. 1 × 10^6^ conidia/mL were inoculated in 20 mL MM supplemented with 5% glucose and different concentrations of Dox in 100 mL Erlenmeyer flasks, and cultivated at 30 °C and 220 rpm on a horizontal shaker for 72 h. 1 mL of supernatant was flash frozen in liquid nitrogen for total protein quantification using a Bradford assay.

The remaining culture was analysed using an Olympus szx7 stereomicroscope connected to a Canon DS126251 camera. For image capture, approximately 5 mL of culture volume was decanted into a 25 mL petri dish. Morphologies were gently agitated with a pipette tip to ensure pellets were physically separated. For each sample, triplicate images were captured from randomly assigned regions of the petri dish. Images were captured on a black background with lighting from above to illuminate fungal pellets.

To determine fungal biomass after imaging, cultures were filtered through triple layered muslin gauze, washed in sterile water, pat dried between paper towels, and added to pre-weighed falcon tubes. Biomass was incubated at 50–65 °C until dry (minimum of 24 h) after which dry weight was determined.

### Automated image analysis

**Fig. 1:**
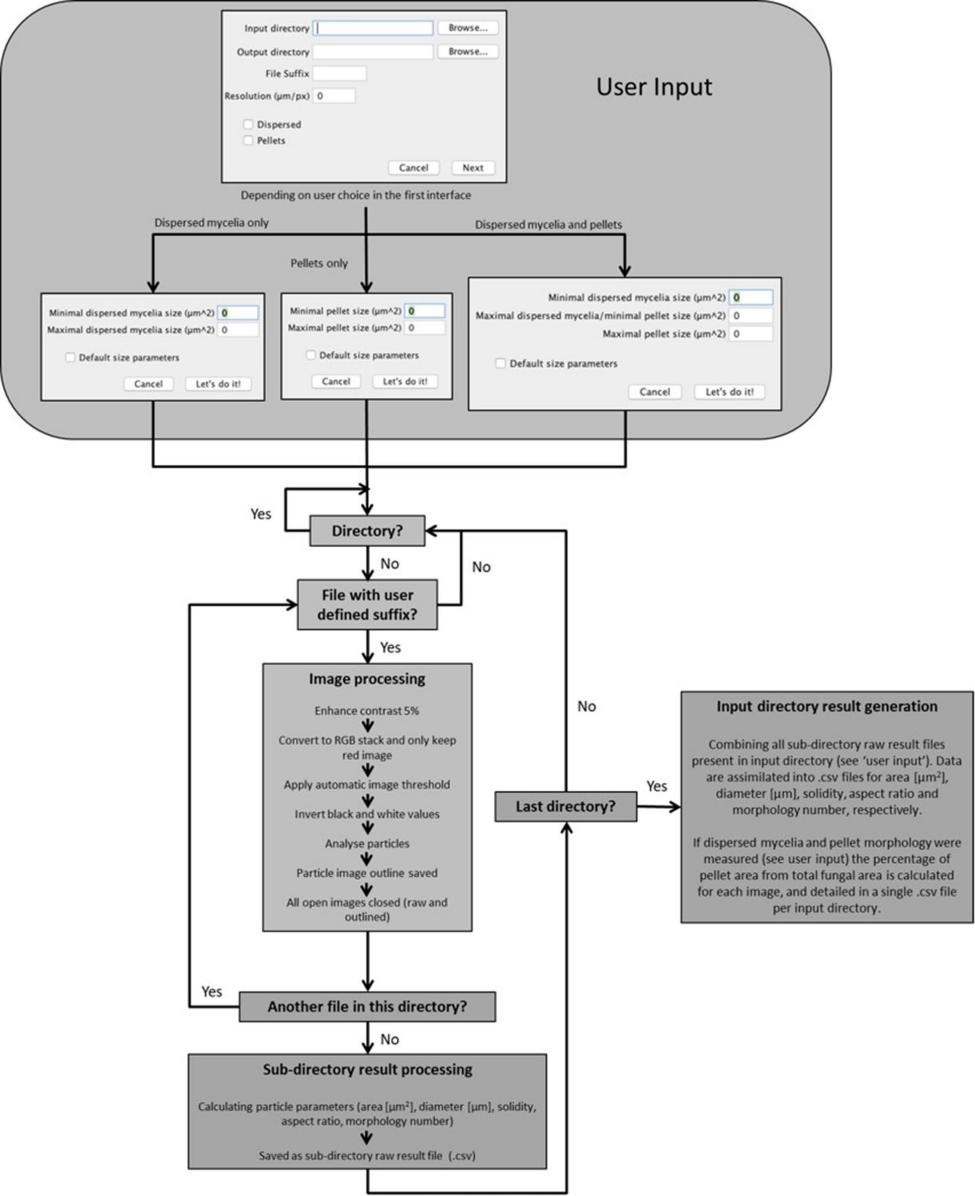
Schematic representation of the image analysis workflow. Users initially (i) define the µm/pixel ratio; (ii) specify the required file suffix (e.g., JPEG); (iii) define the input directory containing all required raw images; (iv) specify a desired output directory, and (v) select whether to analyse pellets, dispersed mycelium, or both morphologies. In all instances, definitions of fungal structures into either dispersed/pellet morphologies are based on area (µm^2^), with a minimal cut-off to remove artefacts that fall below user-specified definitions. If required, default parameters can be used (see main text). All files with the required suffix are analysed in the input directory. Note that the pipeline is compatible with sub-directories, and will calculate raw data files (.csv) for every such folder contained in the input directory. Raw data files contain all pellet/dispersed measurement data (e.g., diameter and aspect ratio) for images contained in the respective sub-directory. Once all images/sub-directories have been analysed, results are generated at the level of input directory (i.e., for every image contained in this folder, irrespective of whether it is divided into a sub-directory). This result file has all parameters for pellet and/or dispersed morphologies extracted into respective .csv files (e.g., diameter, aspect ratio, etc.). Note that, for simplicity, quality control images consisting of the indexed outline of the fungal structure (Fig. 2) are saved in the respective sub-folder of the input directory. Finally, if both pelleted and dispersed morphologies are analysed, the percentage of pelleted morphologies (µm^2^) is calculated as a function of total fungal area (µm^2^), thus, giving a measurement of pelleted and dispersed growth in each image. This latter measurement is recorded for all sub-folders in the input directory, and is saved as a single .csv file

Contrast of raw images (e.g., jpg) was enhanced by 5%, which were then converted to RGB (red–green–blue) stacks. Next, the red image was retained and green/blue deleted. A standard threshold was applied to all images. Note that this threshold limit was selected based on manual interrogation of several hundred images ranging from 10 to 50× magnifications to accurately call pelleted/dispersed morphologies but also omit artefacts. Subsequently, image colour was inverted, and the ‘Analyse Particles’ ImageJ function applied. Images depicting indexed outlines of fungal macromorphologies for pelleted or dispersed morphologies were generated for every raw image (Fig. 1). Output .csv files with the following parameters were calculated for each fungal structure: (i) area (µm^2^), (ii) Feret’s diameter (maximum diameter of each structure, µm), (iii) aspect ratio (maximum diameter/minimum diameter), and (iv) solidity. This latter parameter is derived from two area calculations: first, the observed area of the structure, and second, the hypothetical area that would be occupied if the entire perimeter of the structure was convex. Solidity is calculated by dividing the hypothetical convex area by the observed area, and is a measurement of particle surface integrity, with convex/smooth shapes having a solidity value close to 1 and increasing surface irregularity correlated with solidity values decreasing towards 0. Morphology numbers (MNs) were calculated as described by Wucherpfennig et al. [28, 29]; thus:

$${\text{Morphology number}} = \frac{{2 \times \sqrt {\text{Area}} \times {\text{Solidity}}}}{{\sqrt \pi \times {\text{Feret's diameter}} \times {\text{Aspect ratio}}}}.$$

## Results

### Software development for morphology of pelleted and dispersed growth (MPD) image analysis

Software was written in Java as a plugin for ImageJ2/Fiji [46] and is available for either Windows or Mac (Additional file 4). Before running the application, users are able to define µm:pixel ratios for the experiment, ensuring compatibility of the pipeline with user-defined magnification (Fig. 1). All images are processed by the initial conversion to binary format, after which an automatic threshold is applied, and particles captured using ImageJ (Fig. 1).

**Fig. 2:**
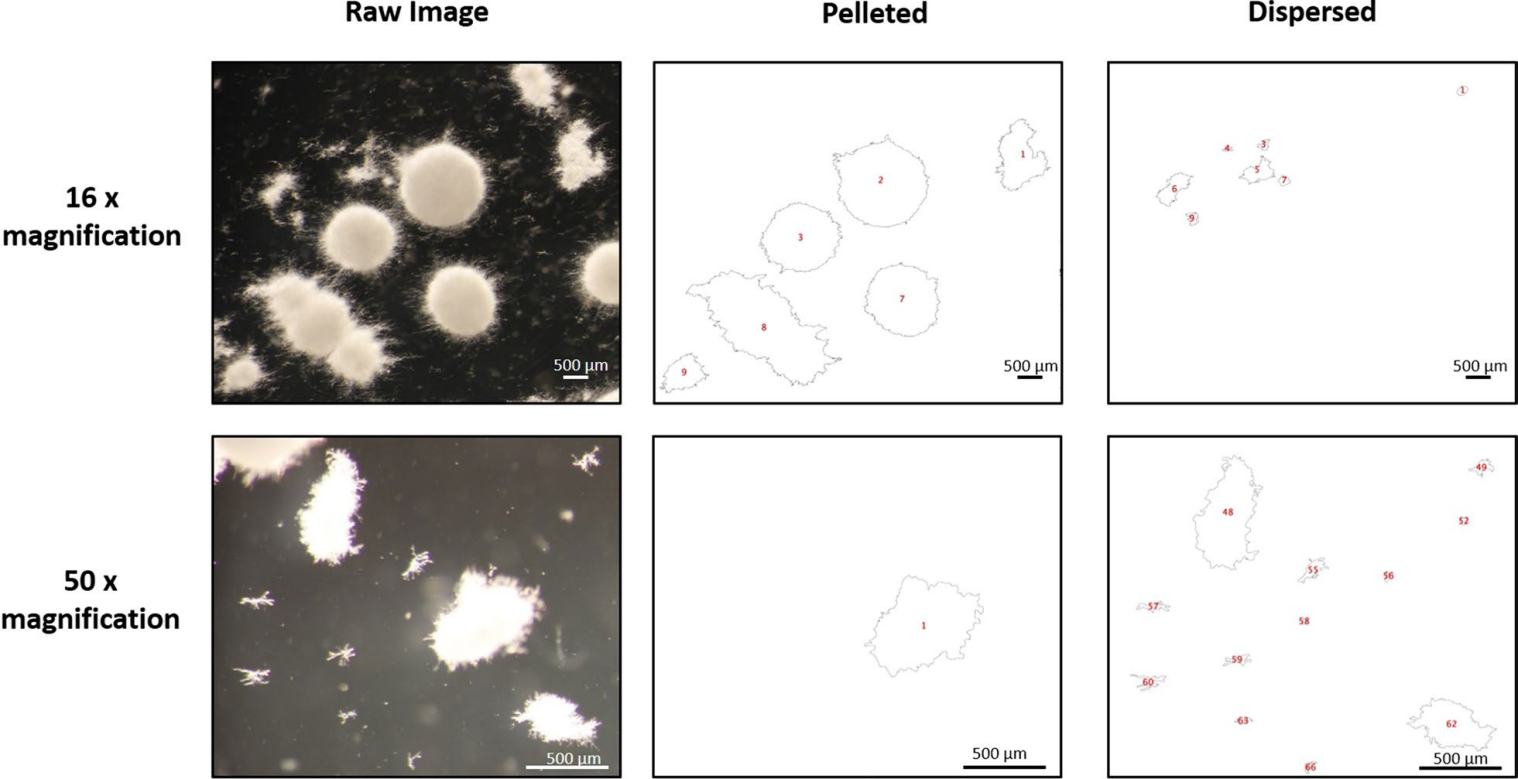
Exemplar automated image analysis of fungal macromorphologies from submerged cultures. 1 x 10^6^ spores/mL of *aplD* conditional expression mutants were grown in MM for 72 h at 30 °C with 220 RPM. Raw images were captured at both 16× and 50× magnification, and subsections of whole images are shown. Scale bars in lower right corners depict 500 µm. For each raw image, two quality control images are generated, in which fungal structures are depicted as outlines indexed with a unique number (red), enabling simple assessment of automated calls by the end user. One outlined image contains pellets and the other dispersed mycelia objects. Note that pelleted or clumped morphologies partially captured at the image edge are excluded from analysis. Processed outlines of fungal structures passing default definitions of pelleted (≥ 500 µm^2^) and dispersed (< 500 µm^2^ and ≥ 95 µm^2^) are shown for 16× magnification. Alternatively, for 50× magnification, pellet sizes definitions were identical, but dispersed mycelium were defined as < 500 µm^2^ and ≥ 20 µm^2^. We found that decreasing the lower size limit (i.e., from ≥ 95 to ≥ 20 µm^2^) enabled accurate automated calls for the dispersed hyphal fragments depicted at higher magnification

Using default parameters of the software, each image is processed twice. First, dispersed morphologies are analysed, which we defined as any fungal structure with an area < 500 µm^2^ and ≥ 95 µm^2^. Second, pellets are assessed, which we defined as any structure with an area ≥ 500 µm^2^. All reported objects from the image with an area < 95 µm^2^ are considered artefacts, and were removed from all analyses. These definitions were made from careful visual inspection of multiple *A. niger* growth phenotypes from all available culture conditions under the magnification (×10) described in this study. Note that the specific size parameters which are used to differentiate between dispersed, pelleted growth, and artefacts can be defined by the user prior to running the software, further ensuring that the pipeline can be used for various magnifications or different fungal species (Figs. 1 and 2). In addition, if submerged culture results highly heterogeneous growth consisting exclusively of pellets or dispersed morphologies, users can select to exclusively analyse the desired morphology (Fig. 1). For default analysis of both dispersed and pelleted morphologies, area, Feret’s diameter, aspect ratio, solidity, and morphology number are automatically calculated for all fungal structures and detailed as a .csv file in a user-specified output directory.

Next, a quality control image is generated for every individual image which has been analysed which is saved in the respective sub-folder that contains the original raw image (Figs. 1 and 2). All values in the respective .csv file (e.g., diameter, aspect ratio, etc.) are ordered in ascending rows based on the numerical value given in the image file, making it simple for the user to visually inspect images and confirm that automated calls of dispersed/pelleted growth are sufficiently accurate for their experimental purpose. We routinely used this quality control aspect to remove inaccurate pellet or dispersed calls (~ 1% of fungal structures in this study).

For each directory, total fungal area from all images is calculated, and the percentage of pelleted growth from this total is reported to give a simple quantitative read out of the relative abundance of pelleted and dispersed growth (Fig. 1). This measurement enables heterogenous fungal cultures containing both dispersed and pelleted morphologies to be rapidly quantified. Prior to validation of MPD image analysis using strain engineering, we cultivated isolate MA70.15 (Table 1) in shake flask cultures routinely used to model either protein or citric acid production (Additional file 5). This analysis demonstrated statistically significant differences in pellet morphology number, area, aspect ratio and solidity between conditions (Additional file 5), indicating that MPD image analysis is an effective method to quantify fungal growth during submerged culture.

### Selection of a target gene that impacts the morphology of *A. niger* based on co-expression network analysis

To further test the quantitative image analysis pipeline, we generated an *A. niger* mutant with defective filamentous growth and pellet morphology during submerged culture. Research in filamentous fungi has revealed that endocytosis has significant roles in filamentous growth [34]. During fungal endocytosis, cargo is received from the plasma membrane in endosomes, and either recycled back to the fungal surface, possibly via the trans-Golgi, or transported for degradation to the vacuole [34]. In addition, endosomes are likely required for transport mRNA and ribosomes throughout the cell [47]. However, the application of controlling endocytosis to optimize fungal morphology during industrial fermentation has not yet been explored.

**Fig. 3:**
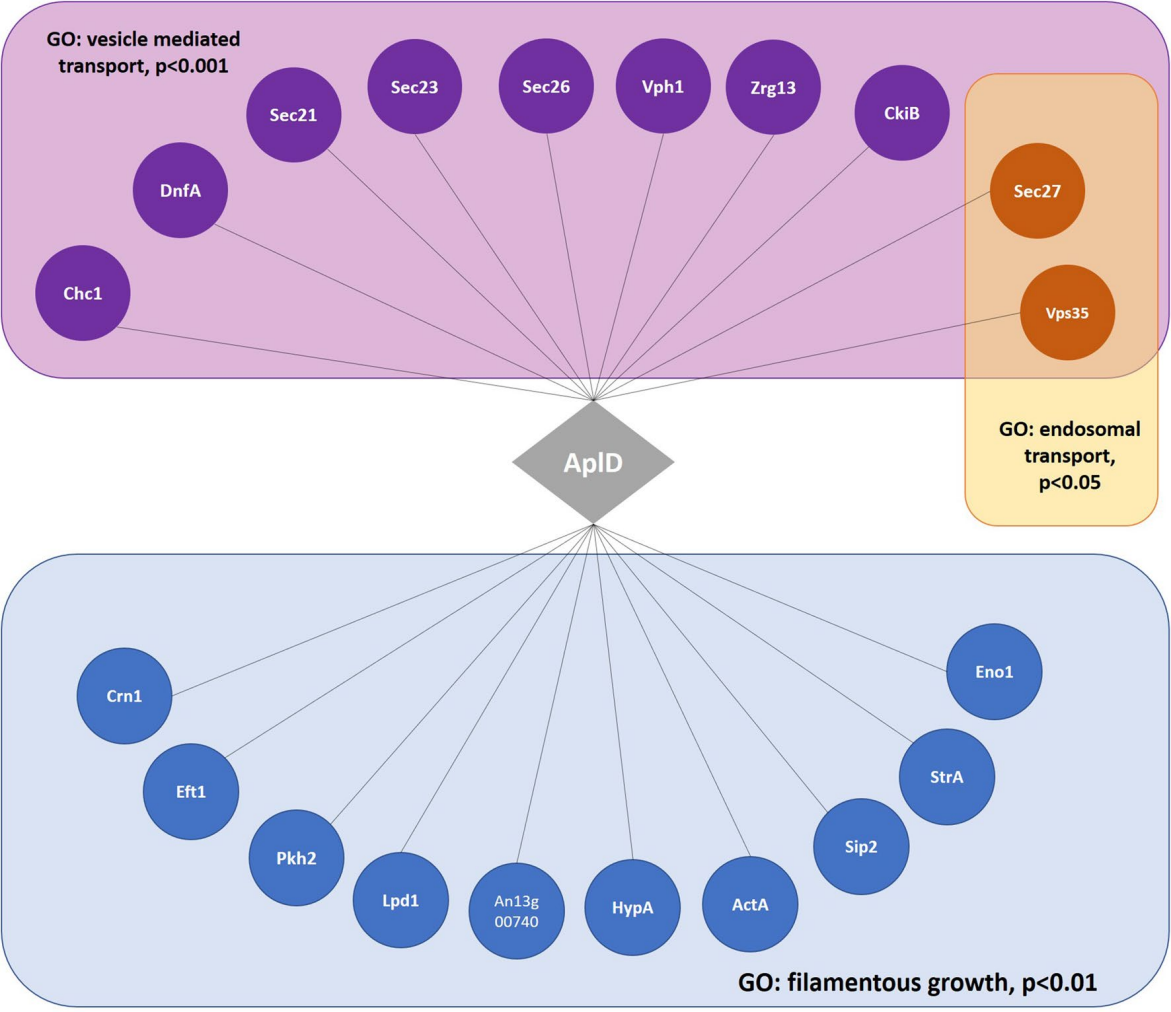
Co-expression network for predicted *aplD* in *A. niger* reveal association of this gene with vesicle-mediated transport, endosomal transport, and filamentous growth. The gene predicted to encode AplD is depicted as a grey diamond, with co-expressed genes depicted as coloured circles. Grey lines indicate co-expression co-coefficients above a Spearman cut-off of 0.7 between *aplD* and respective gene [38]. Benjamini–Hochberg false discovery rate corrected *p* values for GO-enrichment relative to the *A. niger* genome are depicted. Gene name nomenclature from *A. niger* is given. Where this is not available, names from the model fungus *A. nidulans* are given, or from *S. cerevisiae.* A single gene, An13g00740, does not have a standard name in any fungal organism. These data suggest that *aplD* is co-expressed with vesicle and endosomal transport proteins, and additionally with multiple genes that play crucial roles in filamentous growth

To concomitantly test the link between endosomal transport and filamentous growth in *A. niger*, and to select a suitable candidate gene for validating the image analysis pipeline, we interrogated publicly available gene co-expression datasets [38], specifically selecting genes which (i) are predicted to impact endosomal transport based on GO annotation (GO:0016197), and (ii) are co-expressed with genes required for filamentous growth. This resulted in gene An01g02600, which is predicted to encode the ortholog of Alp4, the large subunit of the clathrin adapter protein complex in the budding yeast *Saccharomyces cerevisiae*. In yeast and animals, the clathrin-binding protein complex is crucial for trafficking of protein cargo between the trans-Golgi network and endosomes [35, 48, 49]. *A. niger* co-expression sub-networks revealed association of the An01g02600 gene with vesicle-mediated transport, endocytosis, endosomal transport, microtubule processes, and filamentous growth (Fig. 3 and Additional file 6). We, thus, hypothesised that gene An01g02600, which we name *aplD*, is an important component of endosomal transport and filamentous growth in *A. niger*.

To test the role of *aplD* in filamentous growth, we used a CRISPR-Cas9 gene editing approach to generate *aplD* conditional expression mutants. A 20 bp locus of the 5′ UTR of the *aplD* gene was targeted using a sgRNA, and cut using a Cas9 nuclease [43]. We placed a Tet-on conditional expression system [44] immediately upstream of the coding sequence using 40 base pair of homologous sequences as previously described [43]. The Tet-on system in *A. niger* is an attractive strategy for gene functional analysis as (i) it has undetectable expression in the absence the inducer Dox, thus enabling modelling of null mutant phenotypes [30, 44, 50], (ii) can be used to elevate transcription higher than that of the glucoamylase gene, which is conventionally used for over-expression studies in *A. niger* [38], and (iii) is titratable, whereby concentrations of Dox between 0 and 20 µg/mL results in intermediate phenotypes between null and over-expression mutants [30, 50]. Two PCR confirmed *aplD* conditional expression mutants were generated, which were named TC18.1 and TC18.3.

**Fig. 4:**
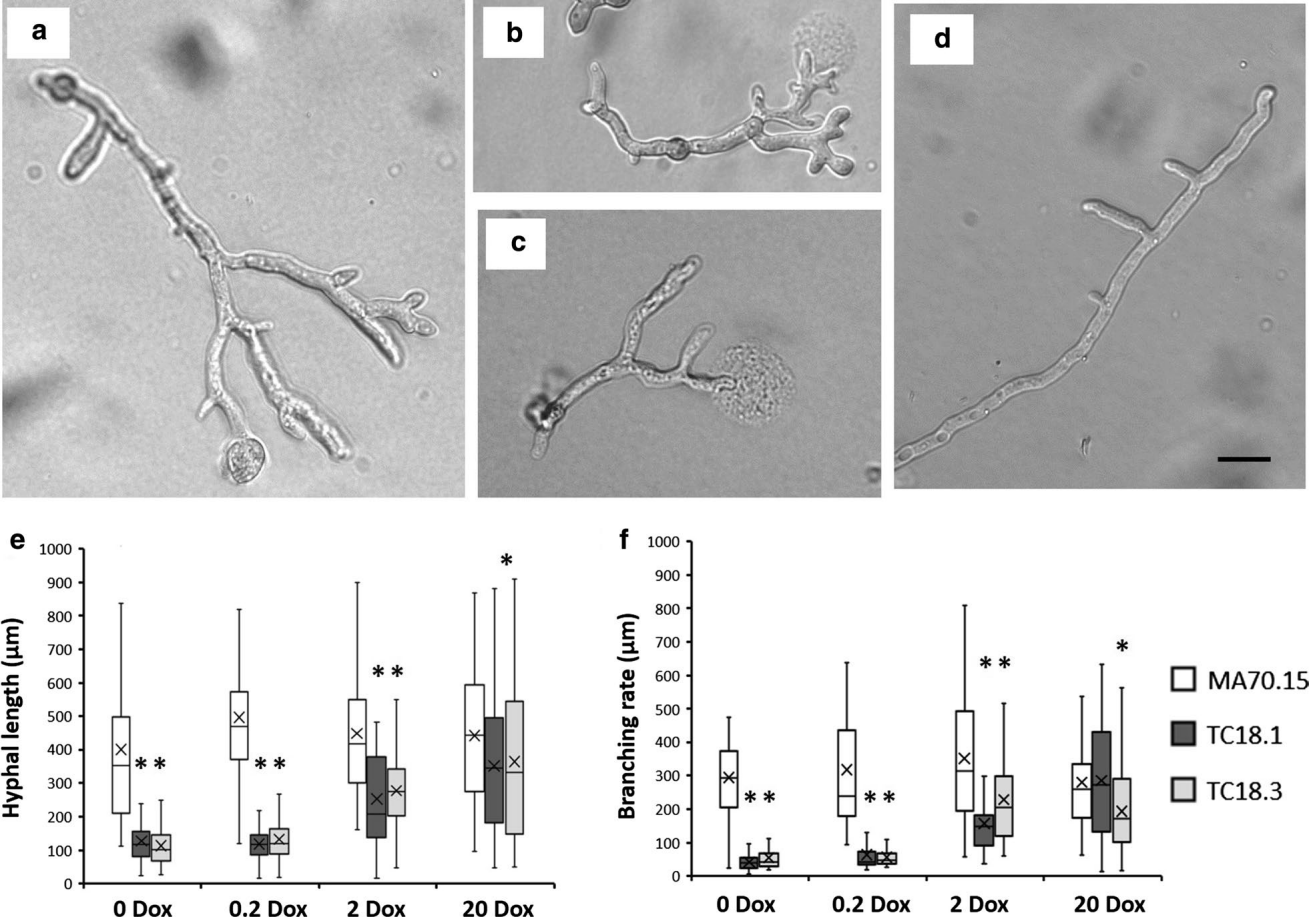
Representative images and quantitative analysis of early hyphal growth in *aplD* conditional expression mutants following titration of gene expression. 1 × 10^4^ spores/mL were inoculated in 10 µl volumes onto MM supplemented with various concentrations of Dox. Plates were incubated at 30 °C in the dark for 18 h. Representative images are shown for technically triplicated experiments. Under 0 and 0.2 µg/mL Dox, TC18.1 and TC18.3 strains showed short, hyperbranched hyphae which often were swollen at the tip (**a**). Under these Dox concentrations, ~ 5–18% of quantified hyphae also demonstrated rupturing at the apex (**b**, **c**). Representative growth phenotype of the MA70.15 control under all Dox concentrations is given in **d**. TC18.1 and TC18.3 hyphal length and branching rate were elevated when expressed using 2 or 20 µg/mL Dox. Box–whisker plots depicting hyphal length (µm, **e**) and branching rate (hyphal length/no. branches, **f**) are given. Asterix indicate highly significant differences between MA70.15 control and conditional expression isolate using a Student‘s *t* test. A minimum of 50 hyphae were analysed per strain/condition. Scale bar depicts 10 µm

We first quantified the impact of *aplD* expression levels on young *A. niger* hyphae. Spores were inoculated on solid MM, grown for 18 h at 30 °C, and length/branch frequency quantified. Under 0 and 0.2 µg/mL Dox, we observed a clear reduction in length and elevated branching in both mutants relative to the control strain MA70.15 (Fig. 4). Interestingly, under these conditions, we also observed swelling at the tip and bursting at the apex (Fig. 4b, c). Hyphal rupturing occurred in ~ 15% and 9% of the analysed mutant hyphae under 0 and 0.2 µg/mL Dox, respectively. This rupturing occurred exclusively at the apical tip, and occurred with what we presume is expulsion of cytoplasm and sub-cellular debris onto the agar surface (Fig. 4b, c). When *aplD* expression was titrated with 2 and 20 µg/mL Dox, hyphal branching and length increased (Fig. 4e, f), and tip rupturing at the apex was not observed. Second, assessment of colony growth on solid undefined complete media and defined minimal media supplemented with various Dox concentrations confirmed a titratable growth defect (under 0, 0.2, and 2 µg/mL Dox) for isolates TC18.1 and TC18.3 relative to progenitor controls (Additional file 7). Mutant isolates produce compact, aconidial colonies that lacked visible hyphae at the periphery. No defects in colony growth were detected when *aplD* was expressed using 20 µg/mL Dox using this assay.

Work in mammalian cell lines has demonstrated that endosomal trafficking increases following exposure to oxidative stress, possibly as a mechanism to increase internalization of cell surface components for repair, storage, or degradation [46]. We thus conducted a simple phenotypic screen to assess the role of *aplD* in oxidative stress by challenging conditional expression mutants with hydrogen peroxide (Additional file 7). Strains TC18.1 and TC18.3 were sensitive to sub-lethal concentrations of oxidative stress (1 mM H_2_O_2_) when expressed with 20 µg/mL Dox. Conversely, these isolates were resistant to a 10 mM lethal dose of H_2_O_2_ when expressed at 0, 0.2, and 2 µg/mL Dox, with mutants grown on 0.2 µg/mL Dox having the strongest resistance phenotype (Additional file 7). These data support the hypothesis that native expression of the *aplD* gene plays an important role in *A. niger* oxidative stress responses.

### Quantitative assessment of *A. niger aplD* conditional expression mutants reveals multiple defects in pellet formation

**Fig. 5:**
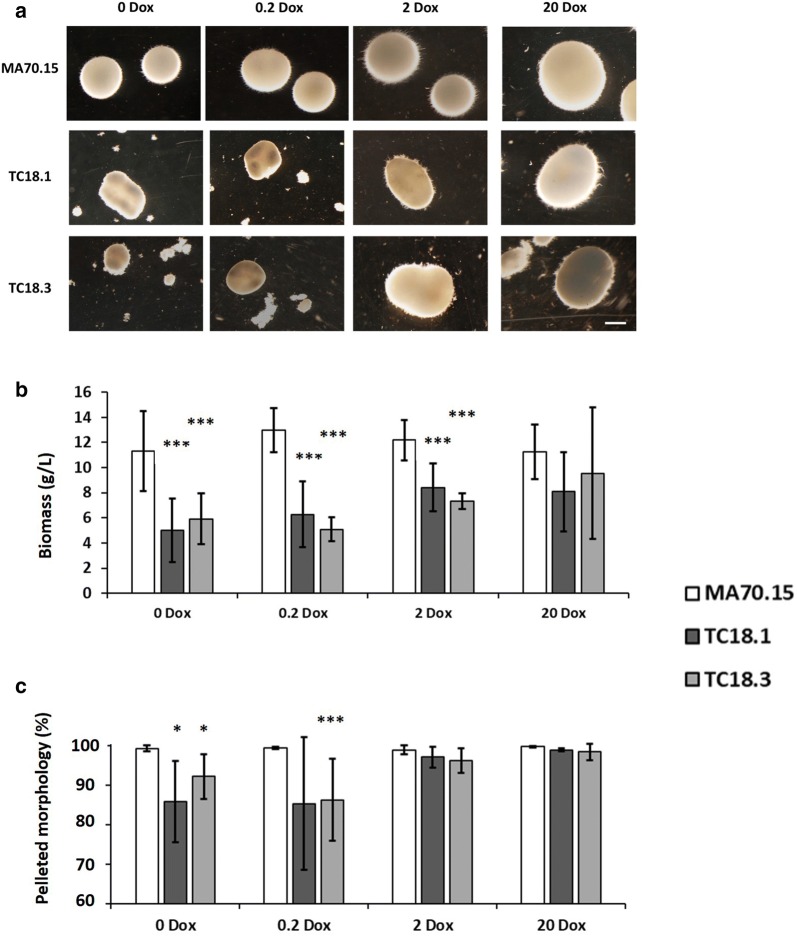
AplD has multiple impacts on *A. niger* submerged growth. 1 × 10^6^ spores/mL of conditional expression mutants and MA70.15 control were inoculated in 20 mL MM with 5% glucose as carbon source and supplemented with various concentrations of Dox. Cultures were grown at 220 RPM, 30 °C, for 72 h. **a** Representative images are depicted for triplicated experiments each consisting of duplicate replicates. Note smaller, irregular shaped pellets and fragments of mycelial growth under 0 and 0.2 µg/mL Dox in mutants TC18.1 and TC18.3. In addition, oblong pellets were observed in these strains under 2 and 20 µg/mL Dox. **b** Dry weight measurements reveal significant reduction in fungal biomass following *aplD* expression using 0, 0.2 and 2 µg/mL Dox. **c** Percentage of pelleted morphologies as a function of the total fungal area measured during image analysis. Note that the expression of *aplD* under 0 and 0.2 µg/mL Dox results in significant decrease in pelleted morphologies, with greater dispersed/clumped morphologies. Error bars report standard deviation from triplicate biological replicates consisting of duplicate technical replicates. Pairwise Student‘s *t* tests were conducted between TC18.1 and TC18.3 relative to MA70.15 control at respective Dox concentrations. *p* values are indicated as (< 0.05, *) and (< 0.01, ***)

Conditional expression mutants and the progenitor control strain were cultivated in liquid MM. Assessment of fungal dry weight revealed reduced biomass in isolates TC18.1 and TC18.3 compared to MA70.15 when grown under 0, 0.2, and 2 µg/mL Dox (p < 0.01, Fig. 5a). This trend towards reduced biomass in mutants was observed in culture supplemented with 20 µg/mL Dox, although this was not statistically significant. These data are consistent with the defects in mutant growth observed on solid media (Fig. 4).

**Fig. 6:**
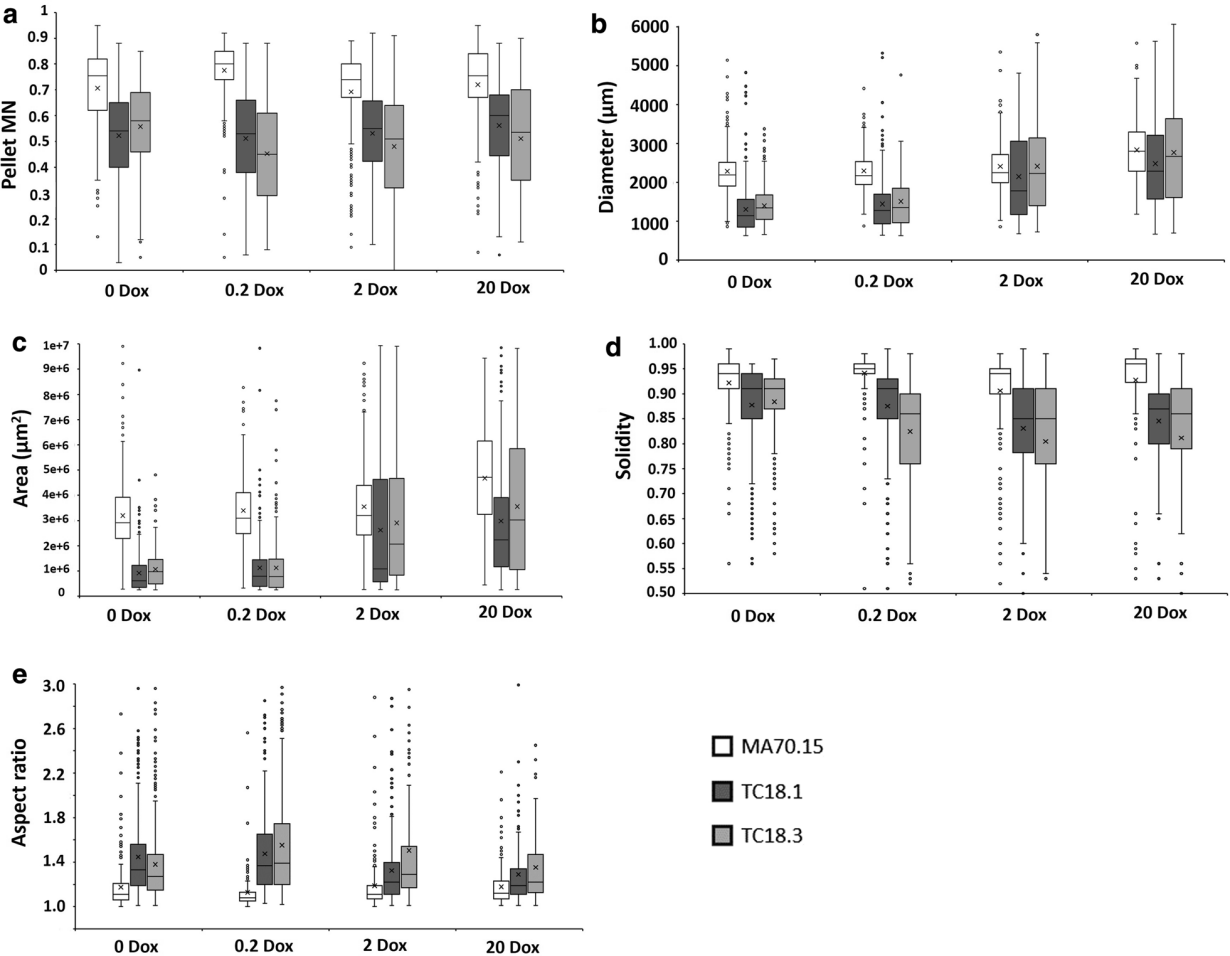
Quantitative analysis of *A. niger* pellet morphology reveals that conditional expression of *aplD* has multiple phenotypic consequences. Shake flask cultures (Fig. 5) were quantitatively analysed using the MPD image analysis pipeline (Fig. 1). Reported are box and whisker plots for pellet morphology number (**a**), diameter (µm, **b**), area (µm^2^, **c**), solidity (**d**), and aspect ratio (**e**). Crosses depict average values. Pairwise Student‘s *t* tests were conducted between TC18.1 and TC18.3 relative to MA70.15 control at respective Dox concentrations. Differences between control and conditional expression mutants were highly significant under all Dox concentrations and for all parameters, with the exception of diameter of strain TC18.3 under 2 and 20 µg/mL Dox. Note that all parameters, with the exception of MN, were titratable using different Dox concentrations with statistically significant increase (diameter and area) and decrease (solidity and aspect ratio) as Dox concentrations were elevated

With regards to quantifying the abundance of both pelleted to dispersed morphologies, our image analysis pipeline demonstrated that MA70.15 reproducibly grew as highly homogenous pellets (Fig. 5b), with > 98.5% of total fungal area qualifying as this growth morphology. In contrast, both *aplD* conditional expression mutants resulted in a decrease of pelleted growth in media supplemented with 0 and 0.2 µg/mL Dox (85–93%, Fig. 5b). In cultures with either 2 or 20 µg/mL Dox, wild-type levels of dispersed morphologies were observed, indicating that sufficient *aplD* expression is necessary for *A. niger* pelleted growth. Further quantitative assessment of submerged culture revealed defects in pellet morphology in isolates TC18.1 and TC18.3 relative to the control. Expression using 0 and 0.2 µg/mL Dox resulted in defects including reduced pellet diameter, area, and solidity, indicating lowered *aplD* expression results in smaller pellets and defects at the pellet surface (Fig. 6). Inspection of the pellet surface using light microscopy confirmed shorter, hyperbranched hyphae in mutant strains under 0 and 0.2 µg/mL Dox when compared to MA70.15.

The role of *aplD* in determining pellet size was supported by a statistically significant increase in pellet diameter and area as Dox concentrations were elevated in mutants TC18.1 and TC18.3 (e.g., between 0.2 and 2 µg/mL Dox, Fig. 6b, c). In contrast, pellet solidity statistically decreased between conditional expression mutants between 0.2 and 2 µg/mL Dox, and between 2 and 20 µg/mL Dox (Fig. 6d). Manual inspection of images for TC18.1 and TC18.3 under 2 and 20 µg/mL Dox revealed that decrease in solidity resulted from intermittent sections of the pellet surface displaying near wild-type levels of hyphal growth (Fig. 5a). As such, reduced solidity in mutant strains under these Dox concentrations may actually represent a closer relationship to the wild-type phenotype than mutant growth under 0.2 or 2 µg/mL Dox, where the pellet surface was generally uniformly defective.

Interestingly, expression of *aplD* using 0 and 0.2 µg/mL Dox resulted in elevated pellet aspect ratio (Fig. 6e), indicating that pellets also more oval than round when compared to the progenitor strain. Similarly, growth of mutants under 2–20 µg/mL Dox resulted in a statically significant reduction in pellet aspect ratio when compared to lower Dox concentrations (Fig. 6e). However, under no conditions tested in this study did TC18.1 or TC18.3 display pellet aspect ratios that were comparable to the control. Note that MNs were uniformly reduced in both mutant strains under all *aplD* expression conditions (Fig. 6a). These data suggest that while MNs may be a useful approach for understanding global changes in submerged morphology, these values should be interpreted in the context of other quantitative measurements of pellet parameters (e.g., aspect ratio and diameter).

### Micromorphology and macromorphology are tightly correlated in *A. niger*

**Fig. 7:**
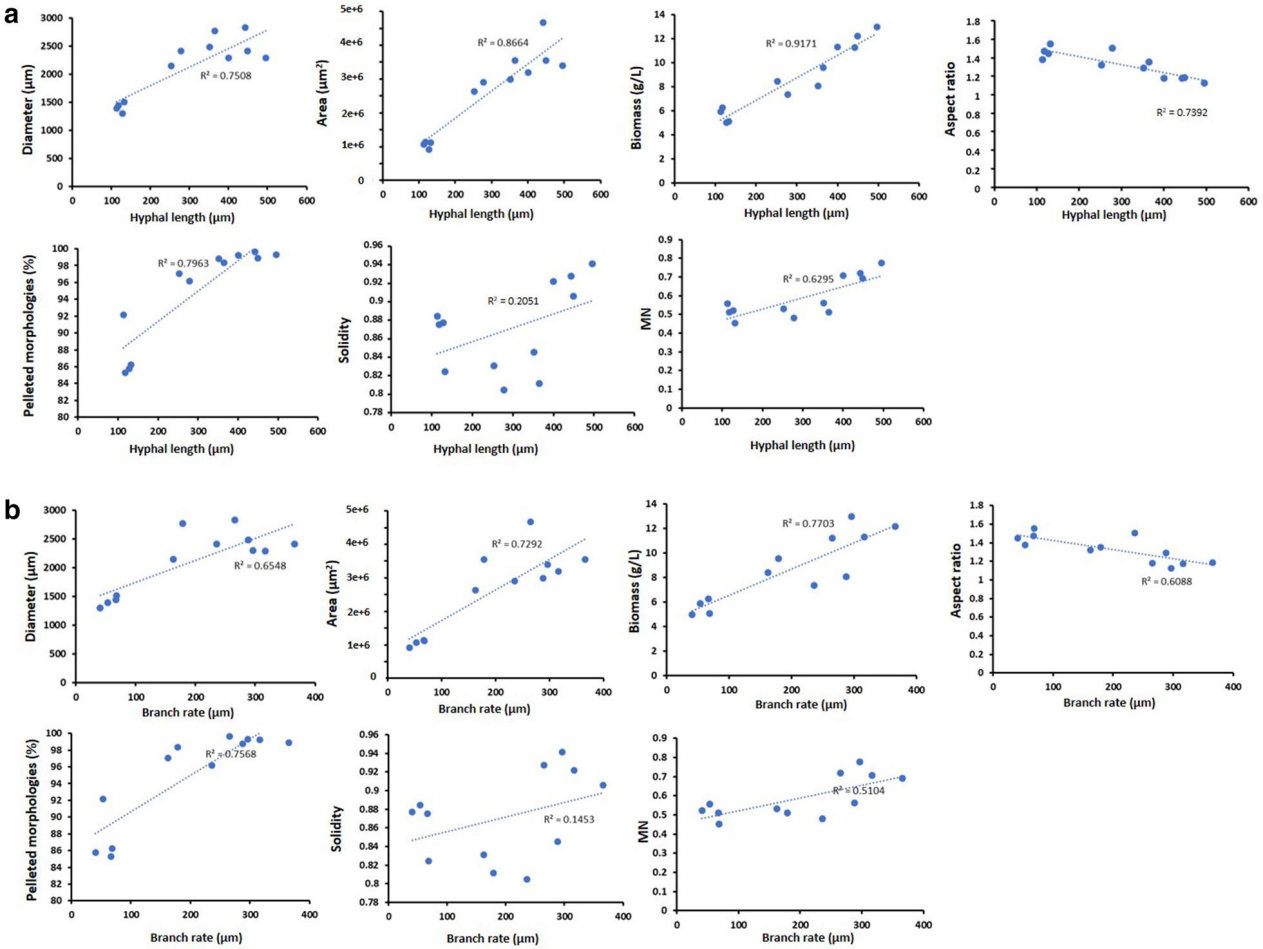
Correlation of hyphal length and branching rate on solid media with submerged pellet parameters. Average pellet parameters from submerged culture were plotted as a function of hyphal length (**a**) and branch rate (**b**) from agar plates (Fig. 4). R^2^ values are shown, with linear line of best fit depicted with a dotted blue line

To assess the connection between filamentous growth phenotypes, i.e., *A. niger*’s micromorphology (Fig. 4) and its macromorphology during submerged culture (Fig. 6), we plotted pellet parameters and biomass as a function of average hyphal length and branch rate (Fig. 7). Both hyphal length and branch rate were mainly positively correlated with pellet parameters and biomass (Fig. 7). For example, an increase in average hyphal length from 100 to 300 µm on solid agar (Fig. 4) was correlated with an increase in diameter from 1500 to 2000 µm (R^2^ 0.75), an increase in pellet area from 1 × 10^6^ to 2.5 × 10^6^ µm (R^2^ 0.86), and an increase in culture biomass from 5 to 8 g/L (R^2^ 0.91). While it is difficult to assess if length or branch rate was more important for pellet parameters, it should be noted that length was more highly correlated with pellet parameters than branch rates, suggesting that hyphal length may be a more important determinant of submerged macromorphology. Note that only pellet solidity was poorly correlated with hyphal morphology. Overall, these data clearly demonstrate that micromorphology and macromorphology are tightly correlated in *A. niger*, offering an avenue for rational engineering of its macromorphological characteristics based on genetic control of hyphal length and branching frequency.

### *AplD* may impact total protein secretion in *A. niger* submerged cultivations

**Fig. 8:**
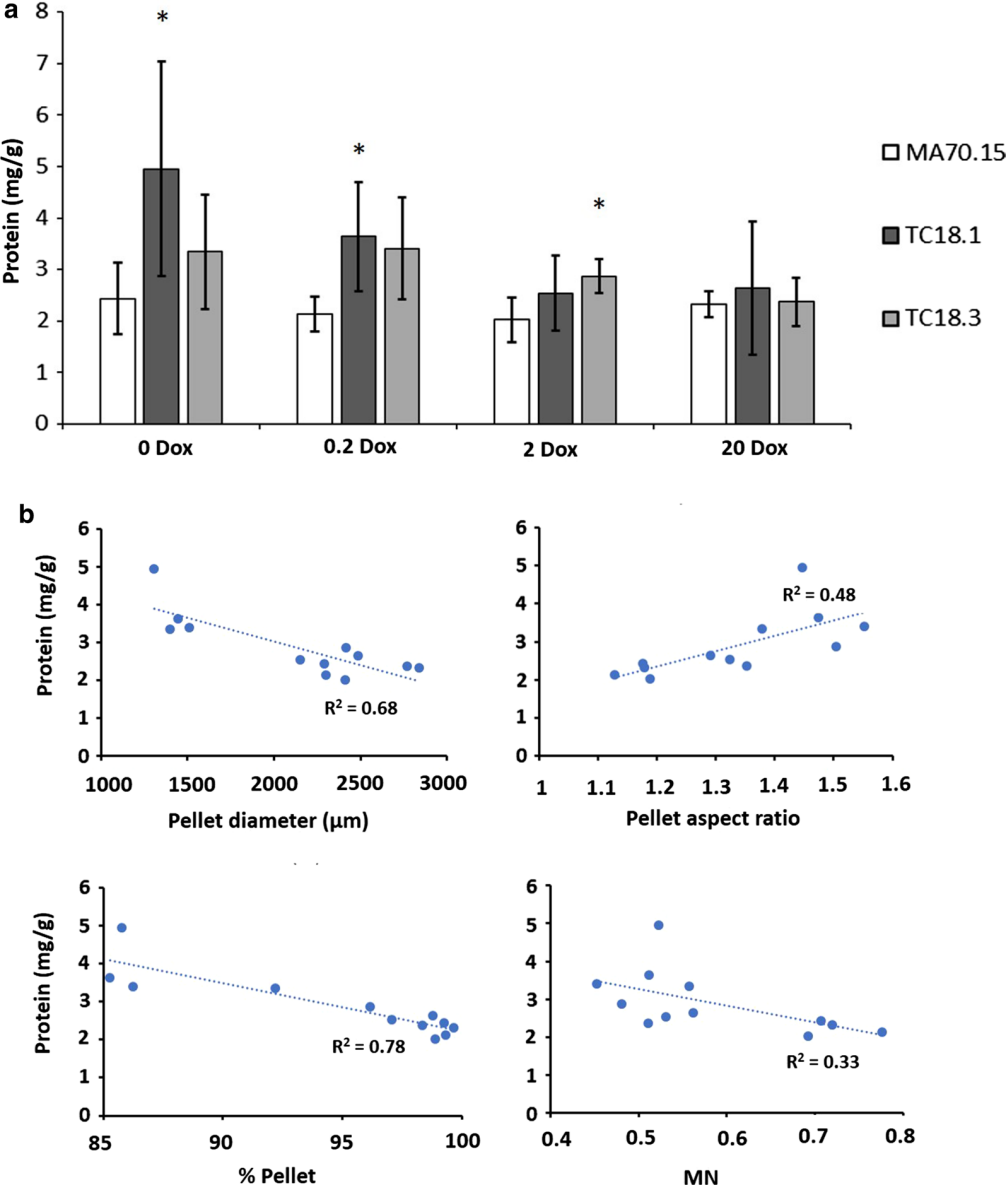
MPD analysis enables correlation between morphological parameters and *A. niger* protein secretion. **a** 1 × 10^6^ spores/mL of conditional expression mutants and MA70.15 control were inoculated in 20 mL MM with 5% glucose as carbon source and supplemented with various concentrations of Dox. Cultures were grown at 220 RPM, 30 °C, for 72 h. Total secreted protein (mg) was determined from culture supernatants using a Bradford assay, which was normalised to fungal biomass (g dry weight). Error bars report standard deviation from triplicate biological replicates, each consisting of duplicate technical replicates. Pairwise Student‘s *t* tests were conducted between TC18.1 and TC18.3 relative to MA70.15 control at respective Dox concentrations. *p* values < 0.05 are depicted with an asterisk. **b** Scatter plots where average total protein secretion (mg protein/g dry weight) was calculated for each strain/Dox concentration and plotted as a function of average pellet diameter, aspect ratio, MN, and percentage of pelleted morphologies present in the culture. Linear lines of best fit (dotted blue lines) are depicted, with R^2^ values shown for each correlation

To interrogate the role of the *aplD* gene in protein secretion, total protein was measured in culture supernatants (Fig. 8a). We observed a decrease in total secreted protein in mutant strains under all Dox conditions, whereby MA70.15 produced protein at concentrations of ~ 25 µg/mL, and TC18.1/TC18.3 produced ~ 18 µg/mL. However, normalisation of total protein (mg) to fungal biomass (g) suggested that mutant isolates might be protein hypersecretors, most notably under 0 and 0.2 µg/mL Dox (Fig. 8a). Plotting protein secretion (mg protein/g dry weight) as a function of various pellet and culture parameters revealed several correlations (Fig. 8b). Specifically, we observed: (i) an increase in pellet diameter from 1.25 mm to 2.5 mm was correlated with a decrease in protein secretion from ~ 4 µg/g to ~ 2 µg/g; (ii) elevated aspect ratio from 1.1 (approximately spherical) to 1.5 (approximately oblong) was positively correlated with protein secretion; (iii) cultures with high percentage of pellets (> 95% of total fungal area) were inversely correlated with protein secretion; and (iv) a possible inverse correlation between average pellet MN and protein secretion was observed (Fig. 8b). These data demonstrate how future strain engineering studies can quantify the relationship between fungal morphology and productivity using MPD image analysis.

## Discussion

In this study, we generated an automated image analysis pipeline for quantification of heterogenous, submerged fungal cultures that consisted of both pelleted and dispersed morphologies (which we call Morphology of Pelleted and Dispersed growth, or MPD analysis). MPD analysis addresses the problem of culture heterogeneity, i.e., those that contain both pelleted and dispersed growth. Using MPD quantification, we were able to rapidly and reproducibly quantify changes following titrated expression of a predicted AplD encoding gene in *A. niger*. From a methodological perspective, MPD is compatible with simple image capture which requires that fungal structures are of light colour with a dark background. Our protocol enables approximately 200 images/day to be captured and analysed per user, which equates to several thousand pellets and several hundreds of thousand dispersed/clumped morphologies.

One potential limitation to MPD analysis is that while the protocol was designed to quantify heterogenous culture of both pelleted and dispersed morphologies, it is currently not applicable to analyse cultures in which large mycelial ‘rafts’ of interwoven, yet dispersed hyphae have formed. Given that our protocol defines pelleted or dispersed morphologies based on area (µm^2^), such large, dispersed mycelia might be erroneously called as pellets. However, it should be noted that these mycelial rafts: (i) were not observed in the growth conditions used in this study; (ii) can be omitted at the quality control stage; and (iii) might be experimentally addressed by dilution of growth cultures prior to image analysis. This latter approach would preserve structural integrity of genuine pellets, while concomitantly diluting dispersed mycelia to smaller fragments for quantification.

In addition, a second possible limitation is that while solidity is assessed as a measure of particle surface integrity, hyphal tip number is not analysed. This latter limitation is because we anticipate that MPD analysis will broadly be used at relatively low magnifications, for high-throughput analysis of fungal growth at the macroscopic level. Our intention is that solidity should be used as a preliminary measure to identify surface modifications between test and control cohorts, which can then be followed up depending on the requirements of the end user. Recent advances in hyphal tip analysis of fungal pellets have been recently been developed [51].

We found that pellet parameters were tightly correlated with growth phenotypes on micromorphological level, specifically hyphal length and branching frequency (Fig. 7). Notably, pellet aspect ratio seemed to be inversely correlated with hyphal length and branch rates. These data further highlight the utility of the MPD program, whereby quantitative measurements of pellet morphology can be correlated with related growth phenotypes to better understand biotechnologically relevant growth.

In addition, we measured total protein abundance in culture supernatant, which identified a putative increase in protein secretion in the *aplD* mutants during expression with 0, 0.2, and 2 µg/mL Dox (Fig. 8a). It should be noted that the elevation in protein relative to the progenitor strain was generally small, and therefore, the biotechnological relevance is currently unclear. Scale-up experiments using Tet-on expression of *aplD* mutants in bioreactor cultivation, with validation of protein abundance using proteomic profiling, are required to validate the utility of these strains, which is a future plan in our laboratory. Notwithstanding these limitations, data presented in Fig. 8b highlight how future studies can use the MPD image analysis pipeline to quantify the relationship between fungal morphology and productivity.

With regards to the use of morphology numbers to characterize pellet parameters, Wucherpfennig and colleagues have demonstrated that *A. niger* pellet morphology number is inversely correlated with production of glucoamylase and β-fructofuranosidase [28, 29]. These authors modified osmolarity and added microparticles to culture media to titrate pellet morphology. Despite the different approaches between these and our study (most notably that we used genetic and not abiotic perturbation to titrate *A. niger* growth types), our data support those of Wucherpfennig et al. [28, 29], as we observed that protein secretion among the various strains and Dox concentrations was also inversely correlated with pellet MN (Fig. 8b). However, a possible limitation to MN is demonstrated in Fig. 6, in which the titratable responses to Dox in the conditional expression mutants (diameter, area, aspect ratio, solidity) are not observed for MN. Thus, while MN number is a useful generic measurement of particle morphology, data presented in this study suggest that MNs should be interpreted in the context of all composite measurements.

## Conclusion

In this study, we have developed a simple and user-friendly image analysis software for quantification of submerged fungal culture consisting of both pelleted and dispersed morphologies. To test this approach, we generated a conditional expression mutant in the cell factory *A. niger*, in which a Tet-on titratable gene switch was genome edited upstream of a gene predicted to encode the endosomal transport protein AplD. The *aplD* gene impacted filamentous growth and response to oxidative stress, and pellet formation during submerged cultivation, indicating that this gene, and endosomal trafficking in general, may be used to control fungal morphology during biotechnological applications. The methods and gene functional analysis conducted in this study may ultimately lead to optimized morphological mutants in filamentous fungal fermentation.

## Additional files

**Additional file 1.** DNA primers used in this study.

**Additional file 2.** Nucleic acid sequence depicting the *aplD* locus. The nearest 5’ gene upstream of *aplD*, An01g02590, is denoted in red with the *aplD* sequence highlighted in bright blue. sgRNA target sites are coloured yellow, and 40 bp sequences targeted by the donor cassette are coloured in bright green. Verification primers for PCR based confirmation of *aplD* are depicted in purple.

**Additional file 3.** DNA sequence of donor cassette used to transform MA70.15 containing hygromycin resistance and Tet-on cassettes.

**Additional file 4.** ImageJ/Fiji MPD plugin for Windows and Mac. In order to run the program, download the latest version (https://imagej.net/Fiji/Downloads). Extract File S4, and then copy/paste the subsequent folder into the ImageJ/Fiji ‘Plugins’ folder. MPD can then be accessed in the ImageJ/Fiji ‘plugins’ drop-down menu.

**Additional file 5.** The MPD image analysis pipeline identifies differences in MA70.15 pelleted growth between protein and citric acid cultivation conditions. (A) Representative images of MA70.15 pellet formation in either protein or citric acid shake-flask culture conditions. Scale bar = 2 mm. (B) MPD Image analysis reveals statistically significant differences in pellet morphology number (MN), area, solidity, and aspect ratio. Triplicate technical replicates were conducted for each culture condition, and triplicate images were analysed per replicate. Student’s t-tests were conducted between each condition, and with a *p* value of <0.05 denoted by *.

**Additional file 6.** Functional predictions derived from gene co-expression network analysis support a role of *A. niger aplD* in endosomal transport and filamentous growth. Networks were previously calculated from over 300 micro-array experiments, with co-expression Spearman co-efficient values above 0.7 considered highly stringent and robust to aid gene functional predictions. The *aplD* gene co-expression network passing a co-efficient threshold of 0.7 was retrieved from FungiDB (n = 109 genes), and a selection of enriched GO-terms are reported. GO enrichment in the *alp4* subnetwork cohort was calculated relative to the *A. niger* genome as a whole using default parameters in FungiDB. Enriched GO-terms were considered statistically significant when Benjamini-Hochberg false discovery correction p-value >0.05.

**Additional file 7.** Phenotypic screening of *aplD* conditional expression mutants reveal defects in growth and susceptibility/resistance to oxidative stress following titration of gene expression. Serial spore dilutions were inoculated in 10 µl volumes onto CM or MM supplemented with various concentrations of doxycycline (Dox). Plates were incubated at 30 °C in the dark, and images captured after 3 days (or 7 days for 10 mM H_2_O_2_). Representative images are shown for technically triplicated experiments.

## Data Availability

The data sets used and/or analysed during the current study are available from the corresponding author on reasonable request.

## Abbreviations

CM: complete medium
AP: adapter protein
BLAST: basic local alignment search tool
Cas: CRISPR-associated
CRISPR: Clustered Regularly Interspaced Short Palindromic Repeats
csv: comma-separated values
Dox: doxycycline
Hyg: hygromycin
MN: morphology number
MM: minimal medium
MPD: Morphology of Pelleted and Dispersed growth
sg: single guide
Tet: tetracycline
