# Supplementary material for: A quantitative image analysis pipeline for the characterization of filamentous fungal morphologies as a tool to uncover targets for morphology engineering: a case study using aplD in Aspergillus niger
Source: Biotechnol Biofuels. 2019 Jun 15;12:149. doi: 10.1186/s13068-019-1473-0 (PMC6570962; doi:10.1186/s13068-019-1473-0)
Supplement: Supplementary file 2 — Additional file 2. Nucleic acid sequence depicting the aplD locus. The nearest 5’ gene upstream of aplD, An01g02590, is denoted in red with the aplD sequence highlighted in bright blue. sgRNA target sites are coloured yellow, and 40 bp sequences targeted by the donor cassette are coloured in bright green. Verification primers for PCR based confirmation of aplD are depicted in purple. [file 13068_2019_1473_MOESM2_ESM.docx]

**A quantitative image analysis pipeline for the characterization of filamentous fungal morphologies as a tool to uncover targets for morphology engineering: a case study using *aplD* in *Aspergillus niger***

**Timothy C. Cairns^1,2,^** ^§^**, Claudia Feurstein^1,2,3,^** ^§^**, Xiaomei Zheng^1,2^, Ping Zheng^1,2^, Jibin Sun^1,2^ and Vera Meyer^1,2,3^**

^1^ Tianjin Institute of Industrial Biotechnology, Chinese Academy of Sciences, Tianjin, 300308, People’s Republic of China

^2^ Key Laboratory of Systems Microbial Biotechnology, Chinese Academy of Sciences, Tianjin 300308, People’s Republic of China

^3^ Department of Applied and Molecular Microbiology, Institute of Biotechnology, Technische Universität Berlin, Berlin, 13355, Germany

**Supplementary File S2:** Nucleic acid sequence depicting the *aplD* locus. The nearest 5’ gene upstream of *aplD*, An01g02590, is denoted in red with the *aplD* sequence highlighted in bright blue. sgRNA target site is coloured yellow, and 40bp sequences targeted by the DonR cassette are coloured in bright green. Verification primers for PCR based confirmation of *aplD* are depicted in purple.

gtattgtctgatgtctctgctcaataggatatctggtgctctgagagcaccctttgtgttgctggtggtaaaagtatcgggcgcatgagatgaaatttagctactgttctctaacgtagtagccttattatataccttctatgtccgcataagtatgaccagcctactagtaggaagtgatctgatcagactttagcgtgctcatcctgtattgcattatatacatatcctaccgtgtgattgacattttcctcgagtgcctcatatgctggtaagtgggacagtagccaaggttaagtaaggtatataatatgtaaaatggctgggcatattaacaacccaagcatgttagctcaggggaagagcgccgggctcataacccggaggtccctggatcgaaaccaggacatgctatccaatttcttttttatattttttgtccatcgtcactagtccccacttttgctatatttttcttcttctatataccttcatctcccgtttactatcattattcaccctccaaggcattttcaacaaccccttagagctacataaggagtgataaaaataatttactcgctgttgtagctttaactatccgtgattaagatcactgtaccagcacgtgacgagctggccgcccttgagggatgccaagccggccaacacgcaaaggacgggcgaggctctcgacgactgaccaccgccgcgttgctgctgctgcatccgatcattactccctgaccccgtcgttattatatcctccatatcaccgacccccggattgcgcagactggtctcttcgcatccgttctttttctccgaccctccccttcacgtcaatcctctcccttcttctcacctccccatccgggggtccttgtggccgccATGGCATCCCGTAGGTGTCCTTgtgttgaaccctcccccacattgctatactgctcccatgcccctgtccccggaattcgatcgctaactgtcgccccctctcccgcctcttatatacagTCAAGCAGTTTATCCGGAACGTGCGTTCGGCCAAGACAATCGCCGATGAACGAGCAGTCATCCAAAAGGAGAGTGCGGCCATCCGTGCGTCGTTTAGGGAAGAGAGCCATGATTCGGGCATCCGgttagcattgacaccgccaacctctagcaacactcgaaccagcttctcatttatttgcacagGAGAAACAATGTGGCTAAGCTTCTGTATTTATTCACCCTCGGTGAACGGACACACTTTGGTCAAATCGAATGTCTGAAGCTACTGGCCTCCCACCGTTTCGCCGACAAGCGCCTGGGATACTTGGGGACGATGCTGTTACTGGATGAGAACCAAGAAGTTTTGACGCTGGTAACAAACTCATTGAAGAAgtgagtctgtattcaaggtcggcttagggtctcctagctaatgttttgcagTGACCTTAATCACTCCAACCAATATATCGTCGGCTTGGCCCTGTGCGCCCTGGGCAATATCGCCTCCGTCGAAATGTCTCGAGACCTTTTCCCCGAAGTCGAAAACCTAATGTCTACTGCAAATCCCTATATTCGGAGGAAGGCGGCACTGTGTGCTATGCGTGTATGTCGCAAGGTTCCCGACCTGCAGGAGCATTTCCTGGAGAAGGCAAAGACTCTTCTGTCAGACAGGAACCATGGTGTTCTGCTGTGCGGTTTAACACTCGCGATTGACATGTGTGAAGCTGAGGAGGCGGAAGAAGGCCAGGAGGGGGTGATCGAGATGTTCCGGCCGTTGGCCGGTGGTCTTGTGCGCTCCCTAAAGGGGTTGACCACCTCGGGATACGCTCCCGAACATGATGTCTCCGGTATCACCGATCCCTTCCTCCAAGTGAAGATACTGCGCCTTCTCAGAGTACTAGGAAGAGGGGATGCGGCGACCAGCGAAATGATCAACGACATTCTGGCCCAGGTGGCCACTAACACGGATTCGACGAAGAACGTCGGCAACGCTATTCTCTACGAGGCTGTCCTGACCATTCTCGACATCGAAGCTGATTCAGGATTGAGAGTGCTAGGTGTTAACATTCTTGGAAAGTTCCTGACCAACAAGGACAACAATATTCGTTACGTTGCTCTCAACACGCTGAACAAAGTTGTTGCAATTGAGCCCAATGCCGTCCAGCGGCACCGCAACACCATTCTGGAGTGTCTCCGTGACCCGGATATCAGTATAAGAAGACGTGCCCTTGATCTCAGCTTCATGTTGATCAATGAGAGTAATGTGCGTGTTCTTGTTCGAGAGCTGCTCGCTTTCCTGGAAGTTGCCGACAACGAGTTCAAGCCAGCCATGACGACTCAAATTGGTATCGCTGCTGACCGATATGCCCCCAACAAACGCTGGCACGTTGACACTATTCTGCGAGTACTCAAGCTTGCTGGTGCCTATGTTAAGGAGCAGATACTCTCGTCCTTTGTTCGCCTCATAGCCACCACACCAGAGCTGCAGACCTACTCGGTGCAGAAGCTTTATCTCTCATTGAAAGAAGACATCTCCCAAGAAGGCCTTACCCTTGCTGCTACCTGGGTCATTGGCGAGTACGGTGATAACCTTCTCCAAGGTGGTCAATATGAGGAAGAGGAGCTAGTTAAAGAGGTCAAAGAGAGCGACATCGTTGACCTGTTCACCAACATCCTCAATAGCACATACGCCACGCAAACAGTGGTGGAATACATCACTACGGCCTCTATGAAGCTTACCGTGCGCATGTCTGATCCAGCACAGGTTGAGCGGCTCCGTCGCTTCCTCAGCAGCCGGACTGCCGACCTGAGTGTTGAGATTCAACAGCGGGCTGTCGAGTACGTCAACCTGTTCGGCTATGACCAGATTCGTCGTGGAGTTCTCGAACGCATGCCCCCGCCTGAGATACGTGAAGAACAAAGAGTCTTGGGTGCACCGACCAAGAAGCGTCAAAGCAAAATCCTGAAAGACAAATCCAAGAAGCCTGCCAAGCAGGCTGAGCAGGACATGCTCCTGGACCTCATGGGCGGCGATGCTCCACCGACCAGCCCGACTACCAACGGTTCCCAAAATACGGCTGATCTCTTGGCCGATATCCTCGGCGGCGATTCCAGCTTATCGTCACCTTCTCCCCAACCTGCGCAGAAGGCAGCAGGATCCACCAATACCAGCGCTATCATGGACTTGTTCAACACTAATGGAAGCACGCCCTCTCCCAAACCTGCGGAGCCTGCATCTTCAGGCTTGGATCTTCTAGCAGGCTTGAGCTCTTCTGCCTCGCCCGCACCCACTCCTGCGCCGTCGGCGACTCCTGCTCATACGGCCTTTAACAAGAACGACCTCTCCCTTGCCCTTCAAGTCCAACGTGGTAGCGGTGGAAGTGCGCAGATTCAGGCTCGTTTCCGCAACAATTCAAGCTTTGGCCGCTTCTCCAATGTTGGATTGCAAGCAGCCGTGCCCAAGAGTCAGAAACTACAGCTAAGCGCGATCAACAAGGCTGATCTCGATGCTGGCGACGAAGGTATCCAGATGCTCAAAGTTGCAGCACTAACTGGGgtaagtcaaaaccaaccgcgccttttctaattgcctttgactaatgtcctgcagGCCCTCCCACCTAAGCTCCGCCTTCGTCTCCGTATCACGTATGCCAAGGATGGCTCTGATCCTGTAACAGACCAGGTAGACTGGACCGAGCCGTGAacggtaaccagggccttactttcatgaatccttatattcgccttacagtaacctttcagaccgcccatctaattttattttatcaacccaattattgtactaggagggaggatggaagcaagcaagccaaccaacccttgggaggagactaccagcgggaaaccaacgcctacttgaccattcatagttcctcacattacatacatgcatgttcatacatacccggtccatttctagctctccgttttcagcatttcttcccttcgctatcatgtcttcttcttgccttctatgtaaatgcttggtatgttgctgaggcctaggagtggccggttcccccatatgttggggtctaaaatgaaaatgttcctgtgctctttaccacatttgtctcctactgtagtgataaatgtagctcgcctgtcatagtcaaccagtagaagtagtaagtagcaagtactacttcagcaaccaccttgccatgctcaacatatcgacgacgtgggctcagtcactccgtcctgttcagtcatggacacccactagtagtacgctatagtatatactctactactgtaatatgaagacaagccagcacagcaggcctgagaacagcacacaccactagaattaaaccctgaaagccagaagtggaaatgatgaactcaaaacggtactatatatctcggagttagaagaaatgccatcacggcgttaaaaacaaatgtccggtgagccgtttgtttgcgcttcagtgatgccttgcaatccccaaactaccatgattgtgactatgactgtacccaaagccgggtgagacaaaggattagaagttccacacgtcatcgataataggaatgaggcaaatcgac
