# Supplementary material for: A quantitative image analysis pipeline for the characterization of filamentous fungal morphologies as a tool to uncover targets for morphology engineering: a case study using aplD in Aspergillus niger
Source: Biotechnol Biofuels. 2019 Jun 15;12:149. doi: 10.1186/s13068-019-1473-0 (PMC6570962; doi:10.1186/s13068-019-1473-0)
Supplement: Supplementary file 3 — Additional file 3. DNA sequence of donor cassette used to transform MA70.15 containing hygromycin resistance and Tet-on cassettes. [file 13068_2019_1473_MOESM3_ESM.docx]

**A quantitative image analysis pipeline for the characterization of filamentous fungal morphologies as a tool to uncover targets for morphology engineering: a case study using *aplD* in *Aspergillus niger***

**Timothy C. Cairns^1,2,^** ^§^**, Claudia Feurstein^1,2,3,^** ^§^**, Xiaomei Zheng^1,2^, Ping Zheng^1,2^, Jibin Sun^1,2^ and Vera Meyer^1,2,3^**

^1^ Tianjin Institute of Industrial Biotechnology, Chinese Academy of Sciences, Tianjin, 300308, People’s Republic of China

^2^ Key Laboratory of Systems Microbial Biotechnology, Chinese Academy of Sciences, Tianjin 300308, People’s Republic of China

^3^ Department of Applied and Molecular Microbiology, Institute of Biotechnology, Technische Universität Berlin, Berlin, 13355, Germany

**Supplementary File S3:** DNA sequence of DonR cassette used to transform MA70.15 containing hygromycin resistance and Tet-on cassettes.

CCTTCACGTCAATCCTCTCCCTTCTTCTCACCTCCCCATCgacgttaactgatattgaaggagcactttttgggcttggctggagctagtggaggtcaacaatgaatgcctattttggtttagtcgtccaggcggtgagcacaaaatttgtgtcgtttgacaagatggttcatttaggcaactggtcagatcagccccacttgtagcagtagcggcggcgctcgaagtgtgactcttattagcagacaggaacgaggacattattatcatctgctgcttggtgcacgataacttggtgcgtttgtcaagcaaggtaagtgaacgacccggtcataccttcttaagttcgcccttcctccctttatttcagattcaatctgacttacctattctacccaagcatcgatatgaaaaagcctgaactcaccgcgacgtctgtcgagaagtttctgatcgaaaagttcgacagcgtctccgacctgatgcagctctcggagggcgaagaatctcgtgctttcagcttcgatgtaggagggcgtggatatgtcctgcgggtaaatagctgcgccgatggtttctacaaagatcgttatgtttatcggcactttgcatcggccgcgctcccgattccggaagtgcttgacattggggaattcagcgagagcctgacctattgcatctcccgccgtgcacagggtgtcacgttgcaagacctgcctgaaaccgaactgcccgctgttctgcagccggtcgcggaggccatggatgcgatcgctgcggccgatcttagccagacgagcgggttcggcccattcggaccgcaaggaatcggtcaatacactacatggcgtgatttcatatgcgcgattgctgatccccatgtgtatcactggcaaactgtgatggacgacaccgtcagtgcgtccgtcgcgcaggctctcgatgagctgatgctttgggccgaggactgccccgaagtccggcacctcgtgcacgcggatttcggctccaacaatgtcctgacggacaatggccgcataacagcggtcattgactggagcgaggcgatgttcggggattcccaatacgaggtcgccaacatcttcttctggaggccgtggttggcttgtatggagcagcagacgcgctacttcgagcggaggcatccggagcttgcaggatcgccgcggctccgggcgtatatgctccgcattggtcttgaccaactctatcagagcttggttgacggcaatttcgatgatgcagcttgggcgcagggtcgatgcgacgcaatcgtccgatccggagccgggactgtcgggcgtacacaaatcgcccgcagaagcgcggccgtctggaccgatggctgtgtagaagtactcgccgatagtggaaaccgacgccccagcactcgtccgagggcaaaggaatagagtagatgccgaccggatcgatccacttaacgttactgaaatcatcaaacagcttgacgaatctggatataagatcgttggtgtcgatgtcagctccggagttgagacaaatggtgttcaggatctcgataagatacgttcatttgtccaagcagcaaagagtgccttctagtgatttaatagctccatgtcaacaagaataaaacgcgtttcgggtttacctcttccagatacagctcatctgcaatgcattaatgcattggacctcgcaaccctagtacgcccttcaggctccggcgaagcagaagaatagcttagcagagtctattttcattttcgggagacgagatcaagcagatcaacggtcgtcaagagacctacgagactgaggaatccgctcttggctccacgcgactatatatttgtctctaattgtactttgacatgctcctcttctttactctgatagcttgactatgaaaattccgtcaccagcccctgggttgcggccgctctagaaccctcggctggtctgtcttacacaagatcacacgctttgatctacaatcaccccaagtatggacctcaggcccaccaacgcccagtggaggcacgtatcttgcgccccagaggacggaagggcaagaacacgaaggccattgcaggtgtggctggtattgccgtggaagacttgaatactgtcacctttaccgagcaggactctccggccggccttgcctactttgacgcctctatccccggtggtgctaagtactgggccacccctatccgggcctttgtcgactcggaaggcaagattggcctggcttcttaccgcgctagcgctactgctaaagccccttacggaatcgatagccagaagaagcccggatcgtacagcatctctgacgtggctcgcggcgaccagcgcgtggtcccgagactggatagacagcggaacgccgcagcggagactgaggaggttgcgcgtaacctcatgaagagccttggctcctagaatacaggtgtttcagtcccaagcaatttttgtctcatgtgtcagacatgttctatcttccacttagagagctttgctgtatgattaggtttttggatggtaaatgtatagttattttcgcacttgttctcatttgtttgtgatataagccggctacatctcattctaagtataaattaaattgcaatttcaaagacgtctcagtaattactgatcactataaacctggttttgccataccgtaaggcaacagccatcttcataactaactagtccgttacatatgaaatacataaatggcgcttaataatccagcgcaacgcccgaccgctttgacaggtaagcgcacgtgacattaaatagggcatccacatcagggtagatacgcatcacatgacccgcacatcctaagcgagggacgggtaagttgaaggagcgaaagacatcagaacatcttggacggtttttcgaacatccatccagccagcaacaaaccgccaaaGAATTCACCATGTCTAGACTGGACAAGAGCAAAGTCATAAACGGCGCTCTGGAATTACTCAATGGAGTCGGTATCGAAGGCCTGACGACAAGGAAACTCGCTCAAAAGCTGGGAGTTGAGCAGCCTACCCTGTACTGGCACGTGAAGAACAAGCGGGCCCTGCTCGATGCCCTGCCAATCGAGATGCTGGACAGGCATCATACCCACTTCTGCCCCCTGGAAGGCGAGTCATGGCAAGACTTTCTGCGGAACAACGCCAAGTCATTCCGCTGTGCTCTCCTCTCACATCGCGACGGGGCTAAAGTGCATCTCGGCACCCGCCCAACAGAGAAACAGTACGAAACCCTGGAAAATCAGCTCGCGTTCCTGTGTCAGCAAGGCTTCTCCCTGGAGAACGCACTGTACGCTCTGTCCGCCGTGGGCCACTTTACACTGGGCTGCGTATTGGAGGAACAGGAGCATCAAGTAGCAAAAGAGGAAAGAGAGACACCTACCACCGATTCTATGCCCCCACTTCTGAGACAAGCAATTGAGCTGTTCGACCGGCAGGGAGCCGAACCTGCCTTCCTTTTCGGCCTGGAACTAATCATATGTGGCCTGGAGAAACAGCTAAAGTGCGAAAGCGGCGGGCCGGCCGACGCCCTTGACGATTTTGACTTAGACATGCTCCCAGCCGATGCCCTTGACGACTTTGACCTTGATATGCTGCCTGCTGACGCTCTTGACGATTTTGACCTTGACATGCTCCCCGGGTAACTAAGTAAGGATCCACTAGTACAGCAGAAGAATCTCTCTCCGCTGTTGCTTCAGTGTCTGCCATGCATTAACTTCATCCTACTGTCCTACCCGCAGTACCCATTCACATTTGCGGCAGATACCAGGTTGTTTTCTATTCCCTCGGTTCTTCAGTTCTTCAGATATTACATTACTCGAGAAATTGGCGGCGATGCAGGAGTTTGGTTGGTTTTAATTGTTTCAGTCTCCTTGACCTGATTTGTATGACATGCAATGTTTCGACGGAAGACTATCTCGGTGAATATACGTCATGAATCATGCGGCCGCGCGTATCACGAGGCCCTTTCGTCTTCACTCGAGTTTACCACTCCCTATCAGTGATAGAGAAAAGTGAAAGTCGAGTTTACCACTCCCTATCAGTGATAGAGAAAAGTGAAAGTCGAGTTTACCACTCCCTATCAGTGATAGAGAAAAGTGAAAGTCGAGTTTACCACTCCCTATCAGTGATAGAGAAAAGTGAAAGTCGAGTTTACCACTCCCTATCAGTGATAGAGAAAAGTGAAAGTCGAGTTTACCACTCCCTATCAGTGATAGAGAAAAGTGAAAGTCGAGTTTACCACTCCCTATCAGTGATAGAGAAAAGTGAAAGTCGAGCTCCCCATCTTCAGTATATTCATCTTCCCATCCAAGAACCTTTATTTCCCCTAAGTAAGTACTTTGCTACATCCATACTCCATCCTTCCCATCCCTTATTCCTTTGAACCTTTCAGTTCGAGCTTTCCCACTTCATCGCAGCTTGACTAACAGCTACCCCGCTTGAGCAGACATCACCGTTTAAACaccATGGCATCCCGTAGGTGTCCTTgtgttgaaccctccccca
