# Supplementary material for: A quantitative image analysis pipeline for the characterization of filamentous fungal morphologies as a tool to uncover targets for morphology engineering: a case study using aplD in Aspergillus niger
Source: Biotechnol Biofuels. 2019 Jun 15;12:149. doi: 10.1186/s13068-019-1473-0 (PMC6570962; doi:10.1186/s13068-019-1473-0)

**A quantitative image analysis pipeline for the characterization of filamentous fungal morphologies as a tool to uncover targets for morphology engineering: a case study using *aplD* in *Aspergillus niger***

**Timothy C. Cairns^1,2,^** ^§^**, Claudia Feurstein^1,2,3,^** ^§^**, Xiaomei Zheng^1,2^, Ping Zheng^1,2^, Jibin Sun^1,2^ and Vera Meyer^1,2,3^**

^1^ Tianjin Institute of Industrial Biotechnology, Chinese Academy of Sciences, Tianjin, 300308, People’s Republic of China

^2^ Key Laboratory of Systems Microbial Biotechnology, Chinese Academy of Sciences, Tianjin 300308, People’s Republic of China

^3^ Department of Applied and Molecular Microbiology, Institute of Biotechnology, Technische Universität Berlin, Berlin, 13355, Germany

**Supplementary File S5: The MPD image analysis pipeline identifies differences in MA70.15 pelleted growth between protein and citric acid cultivation conditions. (A)** Representative images of MA70.15 pellet formation in either protein or citric acid shake-flask culture conditions. Scale bar = 2 mm. **(B)** MPD Image analysis reveals statistically significant differences in pellet morphology number (MN), area, solidity, and aspect ratio. Triplicate technical replicates were conducted for each culture condition, and triplicate images were analysed per replicate. Student’s t-tests were conducted between each condition, and with a *p* value of <0.05 denoted by *.

**Parameters of culture conditions:**

Protein cultivation conditions: 1 x 10^6^ spores/ml were inoculated into MM [30] with 5% glucose as carbon source. Isolates were incubated at 30°C, with 220 RPM, for 72 hours, after which images were captured.

Citric acid cultivation conditions: 1 x 10^5^ spores/ml were inoculated into citric acid media (3 g/L (NH_4_)_2_SO_4_, 3 g/L NaNO_3_, 0.5 g/L yeast extract, and 100 g/L sucrose, with the pH adjusted to 2.5 using HCl). Cultures were incubated at 34°C, with 220 RPM, for 96 hours, after which images were captured.


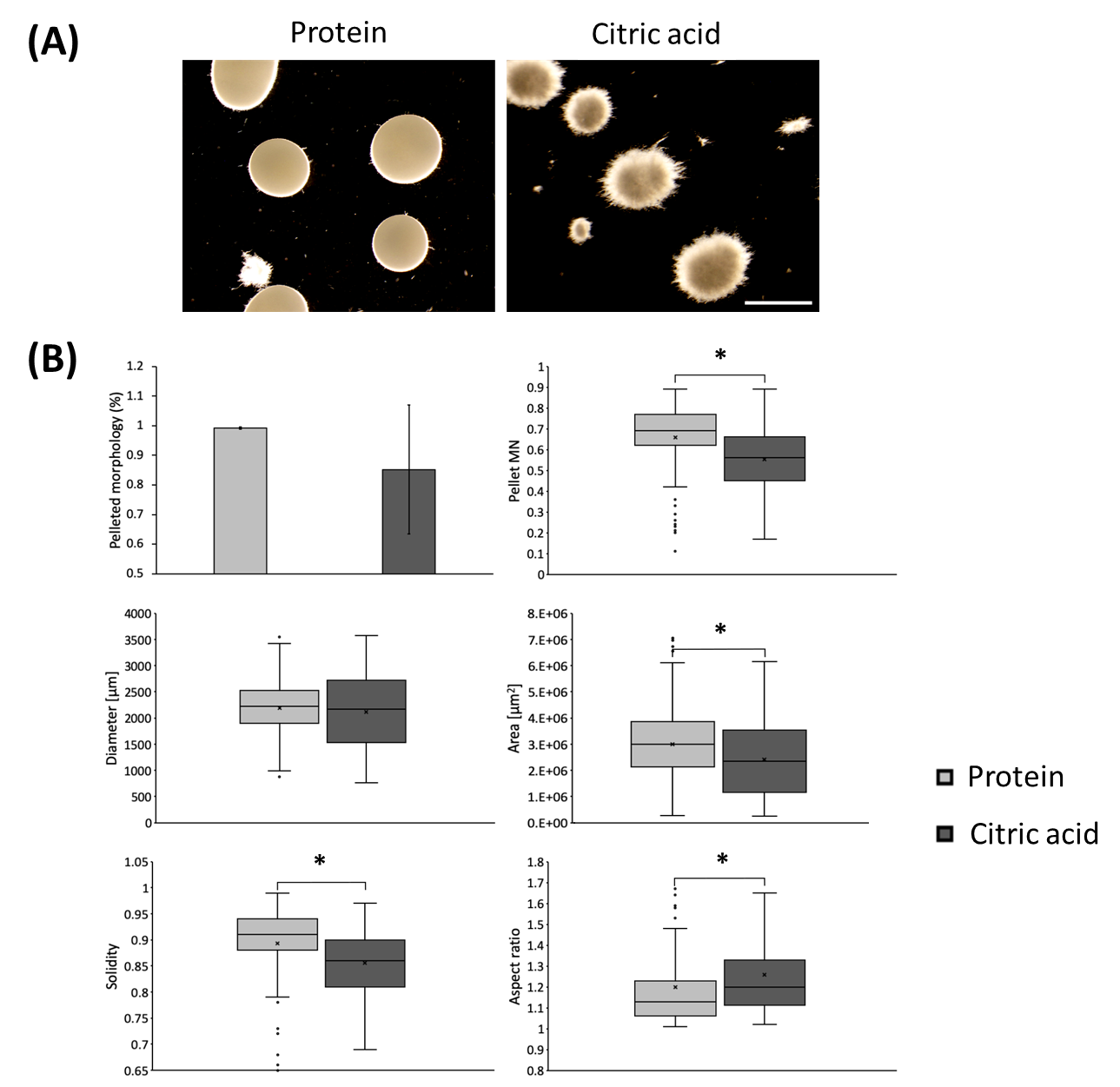

Supplement: Supplementary file 5 — Additional file 5. The MPD image analysis pipeline identifies differences in MA70.15 pelleted growth between protein and citric acid cultivation conditions. (A) Representative images of MA70.15 pellet formation in either protein or citric acid shake-flask culture conditions. Scale bar = 2 mm. (B) MPD Image analysis reveals statistically significant differences in pellet morphology number (MN), area, solidity, and aspect ratio. Triplicate technical replicates were conducted for each culture condition, and triplicate images were analysed per replicate. Student’s t-tests were conducted between each condition, and with a p value of <0.05 denoted by *. [file 13068_2019_1473_MOESM5_ESM.docx]
