# Supplementary material for: A quantitative image analysis pipeline for the characterization of filamentous fungal morphologies as a tool to uncover targets for morphology engineering: a case study using aplD in Aspergillus niger
Source: Biotechnol Biofuels. 2019 Jun 15;12:149. doi: 10.1186/s13068-019-1473-0 (PMC6570962; doi:10.1186/s13068-019-1473-0)

**A quantitative image analysis pipeline for the characterization of filamentous fungal morphologies as a tool to uncover targets for morphology engineering: a case study using *aplD* in *Aspergillus niger***

**Timothy C. Cairns^1,2,^** ^§^**, Claudia Feurstein^1,2,3,^** ^§^**, Xiaomei Zheng^1,2^, Ping Zheng^1,2^, Jibin Sun^1,2^ and Vera Meyer^1,2,3^**

^1^ Tianjin Institute of Industrial Biotechnology, Chinese Academy of Sciences, Tianjin, 300308, People’s Republic of China

^2^ Key Laboratory of Systems Microbial Biotechnology, Chinese Academy of Sciences, Tianjin 300308, People’s Republic of China

^3^ Department of Applied and Molecular Microbiology, Institute of Biotechnology, Technische Universität Berlin, Berlin, 13355, Germany

**Supplementary File S6:** Phenotypic screening of *aplD* conditional expression mutants reveal defects in growth and susceptibility/resistance to oxidative stress following titration of gene expression. Serial spore dilutions were inoculated in 10 µl volumes onto CM or MM supplemented with various concentrations of doxycycline (Dox). Plates were incubated at 30°C in the dark, and images captured after 3 days (or 7 days for 10 mM H_2_O_2_). Representative images are shown for technically triplicated experiments.


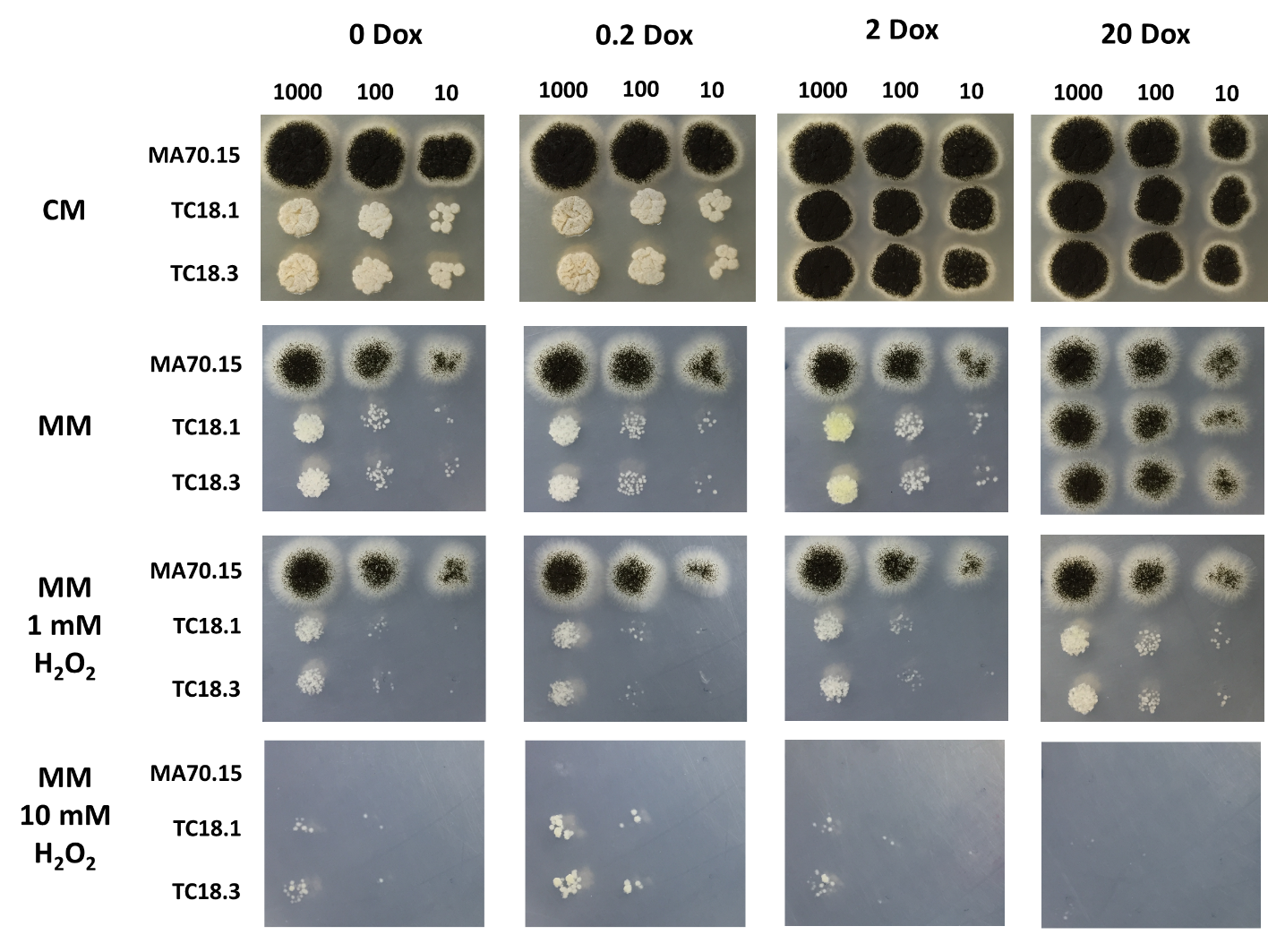

Supplement: Supplementary file 7 — Additional file 7. Phenotypic screening of aplD conditional expression mutants reveal defects in growth and susceptibility/resistance to oxidative stress following titration of gene expression. Serial spore dilutions were inoculated in 10 µl volumes onto CM or MM supplemented with various concentrations of doxycycline (Dox). Plates were incubated at 30 °C in the dark, and images captured after 3 days (or 7 days for 10 mM H2O2). Representative images are shown for technically triplicated experiments. [file 13068_2019_1473_MOESM7_ESM.docx]
